# Characterization of the Bone Marrow Lymphoid Microenvironment and Discovery of Prognostic Immune-Related Factors in Acute Myeloid Leukemia

**DOI:** 10.3390/ijms252313039

**Published:** 2024-12-04

**Authors:** Yoon-Ju Kim, Daehun Kwag, Bo-Reum Kim, Hyunsong Son, Silvia Park, Hee-Je Kim, Byung-Sik Cho

**Affiliations:** 1 Department of Biomedicine & Health Sciences, Graduate Program for Future Medical Research Leaders, The Catholic University of Korea, Seoul 06591, Republic of Korea; rladbswn37@catholic.ac.kr (Y.-J.K.); latias94@catholic.ac.kr (H.S.); 2 Department of Hematology, Catholic Hematology Hospital, Seoul St. Mary’s Hospital, College of Medicine, The Catholic University of Korea, Seoul 06591, Republic of Korea; kdh@catholic.ac.kr (D.K.);; 3 Leukemia Research Institute, College of Medicine, The Catholic University of Korea, Seoul 06591, Republic of Korea

**Keywords:** acute myeloid leukemia, immune checkpoint receptor, T cell exhaustion, γδ T cells, prognosis

## Abstract

Given the limited comprehensive data on the bone marrow (BM) immune environment in acute myeloid leukemia (AML), we analyzed the distribution and phenotype of T cell subsets, including γδ T cells, and their immune checkpoint (IC) ligands on blasts. We performed multiparametric flow cytometry with BM samples taken from 89 AML patients at the time of diagnosis, remission, and relapse/refractory status after chemotherapy and 13 healthy controls (HCs) to identify immune-related risk factors. Compared to the HCs, the T cells of the AML patients exhibited exhausted features including higher TIGIT levels and similar levels of PD-1 and TIM-3. The γδ T cells were exhausted by the upregulation of TIGIT and/or TIM-3 and downregulation of NKG2D and NKp30, with different patterns in the Vδ1 and Vδ2 subtypes. A successful chemotherapeutic response partially restored the exhausted phenotypes of the T cell subsets. The simultaneous analysis of IC receptors on the T cell subsets and their ligands on blasts showed the prognostic value of a specific IC receptor–ligand pair and the feasibility of risk stratification based on their diverse patterns. Our findings clarified the BM T cell landscape in AML, unveiling the prognostic value of γδ T cells in both diagnosis and remission predictions.

## 1. Introduction

Acute myeloid leukemia (AML) originates in the bone marrow (BM), an organ with hematopoietic and immunological systems [1]. AML is characterized by a high degree of molecular and cytogenetic heterogeneity between and within individuals [2,3]. High relapse rates within 3 years and poor post-relapse survival have been observed despite the use of standard treatments such as cytotoxic drugs, hypomethylating agents, and/or allogeneic hematopoietic stem cell transplantation (allo-HSCT), as well as newly developed targeted molecular therapies such as *FLT3*, *BCL-2*, and *IDH1* or *IDH2* inhibitors [4,5,6,7,8]. Recent studies have provided compelling evidence linking immune system dysregulation to AML [9], which has enhanced the research on immunotherapeutic approaches to eradicating clones resistant to the aforementioned treatments and minimized the potential for relapse or the development of refractory AML [10,11,12]. There is increasing evidence that differences in T cell state contribute to the varying prognosis of AML [1]. In particular, regarding T cell exhaustion, several reports have examined the expression of immune checkpoint (IC) receptors on T cells in AML. The ligands for IC receptors, such as programmed cell death receptor 1 (PD-1) and cytotoxic T lymphocyte-associated antigen 4 (CTLA-4), are widely expressed on leukemic cells, thereby inhibiting T cell immune activity [13,14]. Additionally, the expression of T cell immunoglobulin and immunoreceptor tyrosine-based inhibition motif domain (TIGIT) has been reported to be associated with a poor prognosis in AML [15]. Other findings include the involvement of IC receptors such as T cell immunoglobulin mucin 3 (TIM-3) [16], DNAX adhesion molecule (DNAM) 1, CD96, IC receptors that are primarily related to natural killer cells (e.g., NKp30, NKp44, and NKG2D), and OX40. The decreased expression of these receptors has been reported as one of the functional changes in T cells at the time of AML diagnosis [17,18,19]. These receptors have become key targets of research investigating changes in the immune environment associated with AML [20,21]. However, for the reasons outlined below, the features of the lymphoid compartments in AML are debatable [1,9]. Although the BM is the main site of AML development, blood has been used primarily to study the quantity, phenotype, and function of lymphoid cells in the AML immune environment. Furthermore, there is a lack of research on the effects of treatment on BM immune cells. Given the differences in sample type, timing, and methodology, as well as the limited sample size of previous studies, complete profiling of immunological signatures in AML at diagnosis and remission or a relapsed/refractory (R/R) state after therapy remains absent [1]. Furthermore, most investigations focused only on conventional cytotoxic cells, ignoring the potential of unconventional T cells such as γδ T cells, which until now have been rarely studied [22].

γδ T cells are a rare subpopulation that has an extensive repertoire of tumor-sensing mechanisms and may have immunotherapeutic applications in a wide range of tumors [22]. Currently, four main subtypes of human γδ T cells have been documented, which are defined by the TCR δ chain, and the Vδ1 and Vδ2 subtypes are the most predominant [22,23,24]. T cells play key roles in host immunity as primary effectors in response to infection and cancer, preceding the responses of the T cell lineage [22,25]. γδ T cells are characterized by their ability to respond regardless of human leukocyte antigen expression, the production of antitumor mediators, and their high functional plasticity [22]. Given the emerging evidence that γδ T cells show persistent antitumor responses in different types of leukemia and preserve healthy tissues, there have been few reports to characterize γδ T cells in the BM of AML, and the adjacent mechanisms are still poorly understood [22]. Herein, we characterized the BM lymphoid compartment, focusing on different T cell subsets in patients with AML in different disease states who received intensive induction chemotherapy to better understand the immune landscape. The aforementioned IC receptors in the T cell subsets and their corresponding ligands in leukemic blasts were simultaneously examined to identify the optimal immunotherapeutic targets and prognostic immune-related factors.

## 2. Results

### 2.1. T Cell Immune Environments in ND-AML Compared to HC

**Figure 1:**
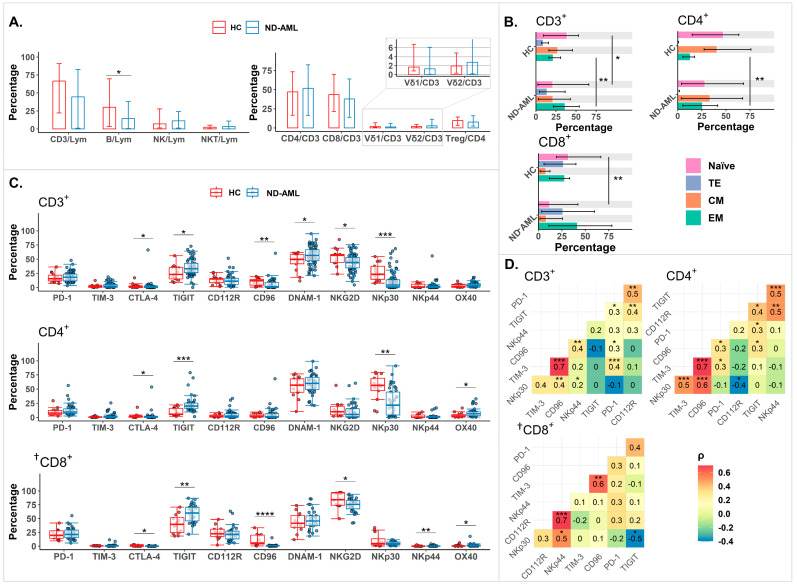
BM T cell immune environment in HCs (N = 13) and ND-AML patients (N = 71). (**A**) Comparison of the frequencies of T cell subsets. (**B**) Comparison of T cell phenotypes in HCs and ND-AML patients. (**C**) Data on T cell proportions expressing ICRs. *p*-values for Figure 1A–C were calculated using Mann–Whitney U Test. (**D**) Correlations among expression of ICRs. Numbers and marks indicate Spearman’s rank correlation coefficient and their significance, respectively. *p*-values of <0.0001 were marked as “****”, ≥0.0001 and <0.001 as “***”, ≥0.001 and <0.01 as “**”, and ≥0.01 and <0.05 as “*”. † CD8^+^ T cells were defined as CD4^−^ T cells among CD3^+^TCRαβ^+^ cells. AML, acute myeloid leukemia; BM, bone marrow; CM, central memory T cell; EM, effector memory T cell; HC, healthy control; ICR, immune checkpoint receptor; Lym, lymphocyte; ND, newly diagnosed; TE, effector T cell.

First, we compared the BM T cells’ characteristics of the ND-AML patients (N = 71) and HCs (N = 13). The BM lymphoid compartment showed a lower percentage of B cells in the patients with ND-AML than in the HCs (*p* = 0.0285; Figure 1A). However, the proportions of the other subsets were similar to those observed in the HCs.

Next, we analyzed the memory states in a subgroup of 10 HCs and 23 ND-AML patients. The ND-AML patients had more effector memory (EM) T cells (*p* = 0.0015) and fewer naïve T cells (*p* = 0.0423) than the HCs (Figure 1B). The increase in EM T cells was more pronounced in the CD4^+^ T cells (*p* = 0.0022) compared to the CD8^+^ T cells. We also investigated how the proportions of T cell memory states varied with age (Supplementary Figure S1). Among these, the TE (effector T) cells within CD3^+^ T cells showed a significant correlation with age (Spearman’s ρ = 0.4350; *p* = 0.0380).

Regarding IC receptor expression (Figure 1C), the ND-AML patients had significantly more TIGIT^+^CD3^+^, TIGIT^+^CD4^+^, and TIGIT^+^CD8^+^ T cells compared to the HCs. However, no significant differences were observed in PD-1^+^ or TIM-3^+^ cells across all the T cell subsets examined. In contrast, the proportions of NKG2D^+^CD3^+^, NKG2D^+^CD8^+^, NKp30^+^CD3^+^, and NKp30^+^CD4^+^ T cells were significantly reduced in the ND-AML patients. Lastly, we examined the correlation between the expression of IC receptors (Figure 1D). Notably, there was a consistently strong positive correlation between the proportions of TIM-3^+^ and CD96^+^ cells across all conventional T cell subsets (0.7331 in CD3^+^, 0.7142 in CD4^+^, and 0.6910 in CD8^+^). Significant positive correlations were also observed between TIM-3^+^ and PD-1^+^ cells and between TIGIT^+^ and PD-1^+^ cells, except for CD8^+^ T cells in the TIGIT and PD-1 correlation. Detailed data on all IC receptors from all T cell subsets are shown in Supplementary Table S1.

### 2.2. Changes in the T Cell Immune Environment with the Disease Status

**Figure 2:**
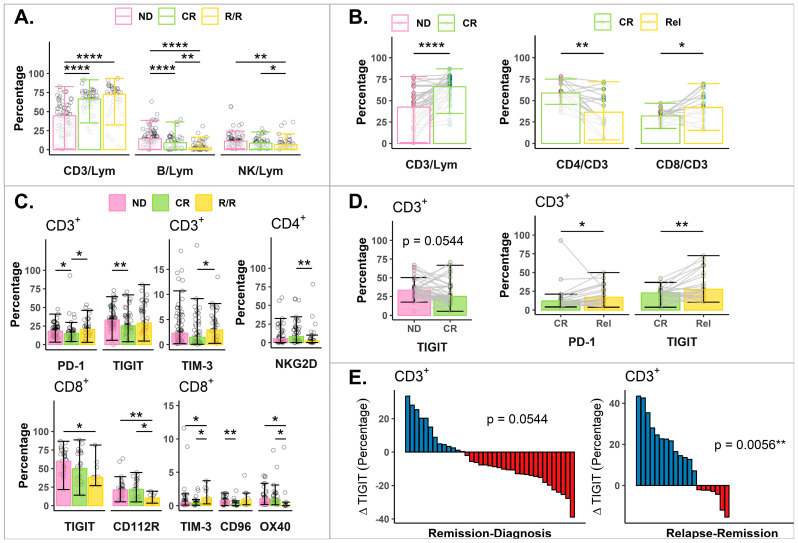
Changes in BM T cell immune environment along with AML disease status (N: ND-AML = 71; CR = 47; R/R = 29; ND-CR pair = 38; CR-Rel pair = 20). (**A**) Subset frequency changes in pooled samples. (**B**) Changes in the frequency of subsets in paired samples. (**C**) Changes in IC receptor expression in pooled samples. (**D**) Changes in IC receptor expression in paired samples. (**E**) Waterfall plot for changes in TIGIT expression at remission from diagnosis. CD8^+^ T cells were defined as CD4^−^ T cells among CD3^+^TCRαβ^+^ cells. *p*-values of < 0.0001 were marked as “****”, ≥0.001 and <0.01 as “**”, and ≥0.01 and <0.05 as “*”. In Figure 2, *p*-values for pooled sample data were calculated using Mann–Whitney U Test and *p*-values for paired sample data were calculated using Wilcoxon signed rank test. AML, acute myeloid leukemia; BM, bone marrow; CR, complete remission; IC, immune checkpoint; Lym, lymphocyte; ND, newly diagnosed; Rel, relapse; R/R, relapse/refractory.

To investigate the changes in the T cell immune environment according to the disease status, we analyzed BM samples collected at the diagnosis (N = 71), CR (N = 47), and R/R (N = 29) stages, including cases of relapse (N = 21) and refractory disease (N = 8). First, in the pooled sample analyses, the frequencies of the T cell subsets were compared based on the treatment response (Figure 2A and Supplementary Figure S2). The CD3^+^ T cell frequencies increased significantly from diagnosis to CR (*p* < 0.0001) and remained elevated at the R/R stage (*p* < 0.0001). This trend was confirmed in paired samples. In paired samples, a decrease in CD4^+^ and a significant increase in CD8^+^ T cells in relapsed patients was also observed (Figure 2B). In contrast, the B cell frequencies declined significantly at all stages, including the CR (*p* < 0.0001) and R/R (*p* < 0.0001) stages. The NK cell frequencies also dropped markedly at the R/R stage (p for ND vs. R/R: 0.0015; p for CR vs. R/R: 0.0258; Supplementary Figure S3 and Figure 2A).

A subgroup analysis of the T cell maturation memory state (23 from the ND-AML group, 23 from the CR group, and 8 from the R/R group) revealed a significant decrease in central memory T cells and CD3^+^, CD4^+^, and CD8^+^ T cell subsets in the R/R group (Supplementary Figure S4). Interestingly, the EM T cells within the CD4^+^ T cells, which were already increased in the ND-AML patients compared to the HCs, increased even further in the R/R group. Conversely, naïve T cells in the CD4^+^ T cells were significantly reduced at the R/R stage compared to the other disease states.

The changes in IC receptor expression across the T cell subsets in the pooled samples are shown in Figure 2C. At the CR stage, the expression of IC receptors decreased compared to that of both the diagnosis and R/R stages. Specifically, PD-1^+^CD3^+^ (*p* = 0.0192) and TIGIT^+^CD3^+^ (*p* = 0.0080) T cells were significantly lower at the CR stage compared to the diagnosis stage, and PD-1 expression was further reduced at the CR stage compared to the R/R stage (*p* = 0.0225). We observed a similar trend in TIM-3 expression on CD3^+^ T cells, with reduced levels at the CR stage compared to the R/R stage (*p* = 0.0167). Significantly lower proportions of NKG2D^+^CD4^+^ and NKG2D^+^CD8^+^ T cells were also noted in the R/R group. These trends were also confirmed through paired sample analyses (Figure 2D). The TIGIT expression was lower at the CR stage compared to the diagnosis stage (*p* = 0.0544), and PD-1^+^CD3^+^ and TIGIT^+^CD3^+^ T cells showed significant increases, while NKG2D^+^CD8^+^ T cells significantly decreased in relapsed patients. The detailed data are presented in Supplementary Tables S2 and S3.

Given the elevated TIGIT expression and its dynamic changes with disease progression, we explored its clinical relevance using paired samples. While TIGIT^+^CD3^+^ T cells exhibited a borderline significant decrease from diagnosis to remission, the relapsed patients consistently showed an increased proportion of TIGIT^+^CD3^+^ T cells compared to the CR patients (*p* = 0.0056, Figure 2E). Interestingly, the patients whose TIGIT^+^ CD3^+^ T cell proportion increased at relapse showed significantly lower overall survival (OS) compared to those patients who did not have an increase in TIGIT^+^ CD3^+^ cells (OS, 3.7 months vs. not reached; *p* = 0.048; Supplementary Figure S5).

### 2.3. Characterization of γδ T Cells in AML and Their Changes with the Disease Status

**Figure 3:**
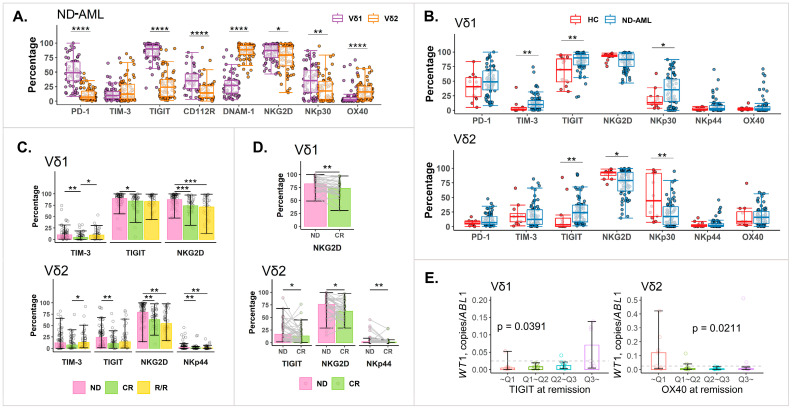
BM Vδ1 and Vδ2 γδ T cells in AML. (**A**) Comparison of IC receptor expression in Vδ1 and Vδ2 T cells in ND-AML patients (N = 71). (**B**) Comparison of IC receptor expression in Vδ1 and Vδ2 T cell subsets in HCs (N = 13) and ND-AML patients (N = 71). (**C**,**D**) Changes in IC receptor expression in Vδ1 and Vδ2 subtypes according to disease status (N: ND-AML = 71; CR = 47; R/R = 29; ND-CR pair = 38; CR-Rel pair = 20). (**C**) Pooled samples. (**D**) Paired samples. (**E**) *WT1* transcript levels based on IC receptor expression in Vδ1 and Vδ2 subtypes. *p*-values of <0.0001 were marked as “****”, ≥0.0001 and <0.001 as “***”, ≥0.001 and <0.01 as “**”, and ≥0.01 and <0.05 as “*”. In Figure 3, *p*-values for pooled sample data were calculated using Mann–Whitney U Test and *p*-values for paired sample data were calculated using Wilcoxon signed-rank test. *p*-values in Figure 3E were calculated using Kendall’s tau test. AML, acute myeloid leukemia; BM, bone marrow; CR, complete remission; HC, healthy control; IC, immune checkpoint; ND, newly diagnosed; Rel, relapse; R/R: relapse/refractory; *WT1*: Wilms tumor gene 1.

We analyzed γδ T cells and their primary subsets, Vδ1 and Vδ2, which are rarely studied in AML patients. First, in the BM of the HCs, inhibitory immune checkpoint (IC) receptors such as PD-1 and TIGIT were significantly upregulated in the Vδ1 subtypes compared to the Vδ2 subtypes. Conversely, the Vδ2 subtypes exhibited higher expression of activation markers like DNAM-1 and OX40 (Supplementary Figure S6). In the ND-AML patients, similar trends were observed. However, the NKG2D and NKp30 expression in the Vδ2 subtypes showed a significant decrease compared to the Vδ1 subtypes (Figure 3A). Comparing IC receptor expression between the HCs and ND-AML patients revealed similar exhaustion patterns to those seen in the conventional T cells (Figure 3B). TIGIT expression significantly increased in both the Vδ1 (*p* = 0.0062) and Vδ2 (*p* = 0.0063) subtypes, while TIM-3 expression increased in the Vδ1 subtypes (*p* = 0.0014). Interestingly, the NKG2D and NKp30 levels were significantly reduced in the Vδ2 subtypes in the ND-AML patients compared to the HCs, while NKp30 increased in the Vδ1 subtypes. The correlation analyses (Supplementary Figure S7) revealed trends similar to those of the conventional T cells. Notable correlations were observed between TIGIT^+^ and other inhibitory IC receptor (PD-1, CD112R, and CTLA-4)-expressing cells, TIM-3^+^ and CD96^+^ cells, and TIM-3^+^ and PD-1^+^ cells.

The analysis of IC receptor expression based on disease status showed distinct trends. Specifically, TIM-3 expression increased significantly at the R/R stage compared to the CR stage in both the Vδ1 (*p* = 0.0038) and Vδ2 (*p* = 0.0193) subtypes, and TIGIT expression significantly decreased at the CR stage compared to that at diagnosis in both the Vδ1 (*p* = 0.0204) and Vδ2 (*p* = 0.0017) subtypes. Additionally, NKG2D expression in the Vδ1 and Vδ2 subtypes and NKp44 expression in the Vδ2 subtypes consistently decreased from diagnosis through the CR to the R/R stage, with significant changes at each stage. The paired sample analysis confirmed these findings (Figure 3D), showing decreased NKG2D expression at the CR stage in both subtypes and reduced TIGIT and NKp44 in the Vδ2 subtypes at the CR stage compared to the diagnosis stage.

We further investigated the relationship between IC receptor expression at the CR stage and measurable residual disease (MRD) levels, which were assessed using *WT1* transcript levels (Figure 3E). In the Vδ1 subtypes, TIGIT expression was positively correlated with *WT1* levels (ρ = 0.3468, *p* = 0.0244), with the highest quartile of TIGIT expression showing very high *WT1* levels. In the Vδ2 subtypes, OX40 expression was inversely correlated with *WT1* levels (ρ = −0.3677, *p* = 0.0141), with very high *WT1* levels observed in the quartile with the lowest OX40 expression.

### 2.4. IC Ligand Expression in Leukemia Blasts and Prognostic Immune-Related Factors

We first attempted to identify differences in OS based on T cell subset frequency at diagnosis, but no differences were observed. Next, we examined IC ligand expressions in blasts to evaluate their potential as prognostic markers. The expression patterns were highly variable among the patients, with no significant differences between the ND-AML and R/R patients (Supplementary Figure S8). Additionally, IC ligand expression in blasts did not demonstrate any prognostic significance.

Focusing on the expression of individual IC receptors in T cell subsets, TIM-3 stood out as a significant prognostic marker for OS. TIM-3 expression was associated with survival in all T cell subsets except the CD4^+^ subset. This finding was validated using a multivariate Cox model, which included covariates such as age, ELN risk groups, and the presence of secondary AML (Supplementary Table S4). CD96, which is strongly correlated with TIM-3 expression, was also identified as a significant predictor of survival.

**Figure 4:**
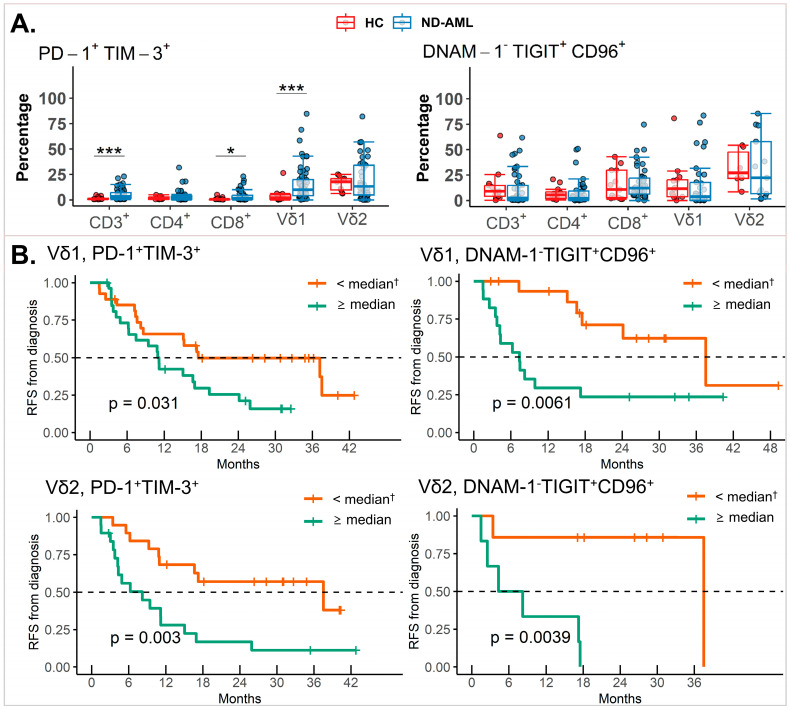
IC receptor co-expression in T cell subsets and their prognostic significance. (**A**) Comparison of PD-1^+^TIM-3^+^ and DNAM-1-TIGIT^+^CD96^+^ proportions in T cell subsets between HCs and ND-AML patients. *p*-values were calculated using Mann–Whitney U Test. (**B**) RFS according to proportion of PD-1^+^TIM-3^+^ and DNAM-1-TIGIT^+^CD96^+^ cells in Vδ1 and Vδ2 subtypes. ≥0.0001 and <0.001 as “***”, and ≥0.01 and <0.05 as “*”. *p*-values were calculated using log-rank tests. † Patients were divided by below/above median percentages of cells expressing PD-1^+^TIM-3^+^ or DNAM-1^−^TIGIT^+^CD96^+^. AML: acute myeloid leukemia; HC: healthy control; IC: immune checkpoint; ND: newly diagnosed; RFS: relapse-free survival.

Using additional gating strategies, we evaluated the frequencies of cells co-expressing multiple IC receptors, such as the PD1^+^TIM3^+^ and DNAM-1^−^TIGIT^+^CD96^+^ subsets. Notably, the frequencies of PD1^+^TIM3^+^CD3^+^, PD1^+^TIM3^+^CD8^+^, and PD1^+^TIM3^+^Vδ1 γδ T cells were significantly higher in the ND-AML patients compared to the HCs. No significant differences were observed in the DNAM-1^−^TIGIT^+^CD96^+^ cell frequencies between the groups (Figure 4A). Interestingly, γδ T cells exhibited a significantly higher frequency of PD1^+^TIM3^+^ cells compared to the other T cell subsets, while DNAM-1^−^TIGIT^+^CD96^+^ cells were uniquely elevated in the Vδ2 subtypes (Supplementary Figure S9). The co-expression of these IC receptors correlated strongly with survival outcomes. For relapse-free survival (RFS), high frequencies of PD1^+^TIM3^+^ or DNAM-1^−^TIGIT^+^CD96^+^ cells were associated with significantly better outcomes when stratified by median expression levels. In the Vδ1 subtypes, the differences in RFS were statistically significant (*p* = 0.031 for PD1^+^TIM3^+^ cells and *p* = 0.0061 for DNAM-1-TIGIT^+^CD96^+^ cells). Similarly, the differences in RFS were also significant in the Vδ2 subtypes (*p* = 0.0061 for PD1^+^TIM3^+^ cells and *p* = 0.0039 for DNAM-1^−^TIGIT^+^CD96^+^ cells) (Figure 4B).

### 2.5. Prognostic Value of a Specific IC Receptor–Ligand Pair and Modeling Their Diverse Patterns

**Figure 5:**
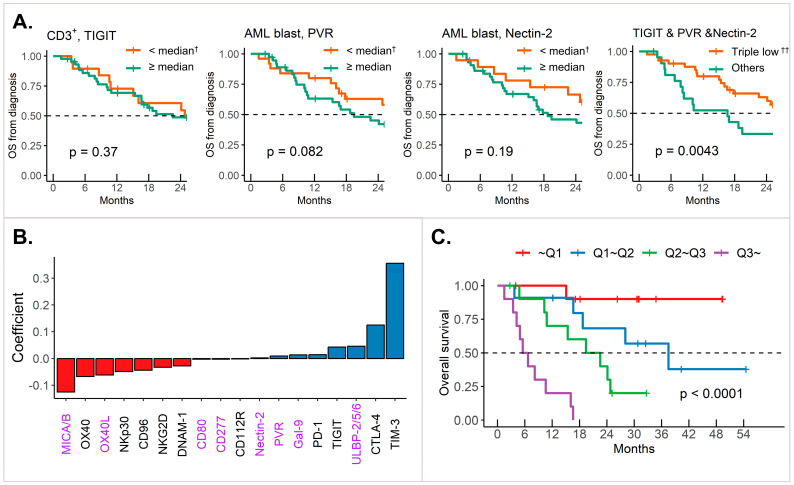
Prognostic value of specific IC receptor–ligand pair and risk stratification model based on their diverse patterns. (**A**) Survival differences based on TIGIT expression in CD3^+^ cells and PVR and Nectin-2 expression on blasts. (**B**) Coefficients of L1 regularized model predicting survival based on IC receptor and ligand expression. Purple indicates Ligand expression on AML blasts, black indicates Receptor expression on T cells. (**C**) OS differences based on model scores. † Patients were divided by below/above median percentages of cells expressing TIGIT, PVR, or Nectin-2. †† Patients were divided into those with concurrent expression of less than the median percentage of TIGIT, PVR, and nectin-2, and others. The dashed line shows the line where the survival rate is 50%. p-values in Figure 5 were obtained using log rank test. AML: acute myeloid leukemia; IC: immune checkpoint; OS: overall survival; Q: quantile.

Given the unveiled prognostic significance of IC receptors and the uncertainty of IC ligands, we hypothesized that the combination of IC receptor expression in T cells and IC ligand expression in blasts could affect prognosis. TIGIT served as a focal clue for this analysis. TIGIT expression alone at diagnosis was not significantly associated with OS. Similarly, the expression of TIGIT’s ligands, poliovirus receptor (PVR) and Nectin-2, on blasts showed no prognostic value when considered independently. However, when we simultaneously applied the cut-offs, which maximized the statistics for TIGIT, PVR, and Nectin-2 expression for survival differences, a clear survival difference emerged. Patients with high expression of TIGIT on T cells and high PVR and Nectin-2 expression on blasts demonstrated a significantly poorer survival rate (*p* = 0.0043, Figure 5A).

In this context, we developed a predictive model based on the expression of IC receptors on CD3^+^ T cells and IC ligands on blasts. This model used an L1 regularization approach to identify key variables. In this model, TIM-3 emerged as the strongest predictor of survival (Supplementary Figure S10). The coefficients of the model using the λ to maximize the C-index are shown in Figure 5B. The model identified the over-expression of inhibitory receptors (TIM-3, CTLA-4, TIGIT, and PD-1) and under-expression of activating receptors (DNAM-1, NKG2D, NKp30, and OX40) as being associated with increased patient hazards in terms of OS. Most ligands exhibited coefficients that aligned with their corresponding receptors, except for ULBP-2/5/6. Among the IC ligands, reduced expression of MICA/B had the most significant negative impact on survival. The model effectively distinguished patient survival when the patients were divided into quartile groups based on the model’s predictions (Figure 5C).

## 3. Discussion

Recent transcriptome data from BM samples have revealed the immune landscape heterogeneity of AML [26]. These transcriptome features can describe the global BM microenvironment of AML; however, detailed phenotypes of each component must be established. In this context, the characteristics of the BM lymphoid compartment in AML are a matter of debate because of the limited comprehensive data on the distribution and phenotype of the T cells, their expression of IC proteins, and their interactions with the corresponding ligands on blasts [1,18]. The current study showed that the distribution of lymphoid cells in ND-AML was significantly altered, mainly due to a reduced number of B cells compared to that in the HCs. The B cell frequency decreased further after chemotherapy. CD3^+^ T cells showed no significant differences in frequency. While there is a discrepancy in the BM T cell frequency compared to previous studies [18,19,20], our data are in line with those of Williams et al. who showed preserved T cell populations using immunohistochemical staining of BM biopsy slides [27]. However, the CD3^+^ T cells in the BM had less naïve and more effector memory subsets than those of the HCs, similar to a recent study in peripheral blood (PB) [28]. After chemotherapy, the CD3^+^ T cell frequency increased significantly during CR, but there was no change in memory phenotypes. Meanwhile, there were no differences in BM Treg frequency in our study, consistent with the findings of the study by Williams et al. [27], in contrast to previous findings that showed elevated Tregs in the PB [1,11] and, in some reports, in the BM of patients with AML [29,30]. Chemotherapy did not change the BM Treg frequency at the CR or the R/R stage, while Williams et al. showed an increased frequency in relapsed AML [27]. We found no correlation between the frequency of CD3^+^ T cells and T cell subsets at the time of diagnosis and the clinical outcomes. These findings did not align with those of previous studies linking the percentages of BM T cells with OS [31,32]. Collectively, these results suggest that the frequency of CD3^+^ T cells and T cell subsets vary significantly and that larger studies are needed to confirm some contradictory results. However, the current study showing the preservation of the T cell subset population in the BM supports the application of T cell-harnessing therapies for AML, such as IC inhibitors and bispecific antibodies [33,34]. However, the wide range of T cell subset frequencies suggests heterogeneity in the BM lymphoid compartment in AML, which may partly explain the modest efficacy of IC inhibitors and immune cell engagers in clinical trials [33,34,35].

The current study revealed varied IC protein expression in T cell subsets and blasts, further supporting the heterogeneity of the BM immune environment in AML. Some studies in BM have shown that T cells display aberrant activation profiles, as well as phenotypic and transcriptional features of exhaustion, compromising an effective endogenous immune response against AML [27,28,36,37,38,39,40]. Given the differences in samples and approaches, many studies have shown increased PD-1^+^ T cell [20,21,28,29,30] and similar TIM-3^+^ T cell [37,39,40] frequencies in ND-AML patients. Our study did not show significant increases in PD-1^+^ or TIM-3^+^CD3^+^ (CD4^+^ and CD8^+^) T cells, consistent with a previous report that did not show differences between the PB and BM [40]. Instead, our data revealed significant increases in TIGIT^+^CD3^+^ (CD4^+^ and CD8^+^) T cells. The high frequency of TIGIT^+^ T cells contrasted with those of TIM-3^+^ and CLTA-4^+^ T cells. Only a few reports have evaluated TIGIT in BM T cells [41,42] despite the high expression of TIGIT in blood T cells from patients [34,35,36]. We observed a significant association between TIGIT and PD-1 in CD3^+^ T cells, consistent with a recent study that showed increased co-expression of TIGIT and PD-1 in BM CD8^+^ T cells [42]. Given the increased frequency of CD3^+^ T cells at the CR stage, the pooled sample analysis showed that the expression of TIGIT or PD-1 on T cells was attenuated in response to chemotherapy, as was shown for PD-1 and TIM-3 in previous studies [28,39,41]. The recovered T cell frequency, along with a partially improved exhaustion, suggests BM immune reconstitution after chemotherapy with a therapeutic response. Moreover, the paired sample analysis showed similar decrease patterns in TIGIT^+^CD3^+^ (CD4^+^) T cells at the CR stage compared to that at relapse. A small group of patients who had more TIGIT^+^ CD3^+^ T cells at the CR stage than those who relapsed had inferior survival, suggesting a potential contribution of exhausted T cells through TIGIT expression to leukemia relapse. This was further supported by a worse survival rate in patients who had more TIGIT^+^CD3^+^ T cells and the corresponding ligands, Nectin-2^+^ and PVR^+^, on blasts. Collectively, our study provides compelling evidence to trigger clinical trials using anti-TIGIT antibodies against AML, similar to many ongoing clinical trials using these antibodies for solid tumors [43].

In particular, we discovered that the TIM-3^+^CD3^+^ (CD4^+^ and CD8^+^) T cell frequency was significantly associated with worse survival, even without differences compared to the HCs. Given the lack of previous reports showing the prognostic significance of TIM-3^+^ T cells in the BM, our data are somewhat consistent with previous reports showing an association between TIM-3^+^ immune cells in the PB and a worse prognosis in AML patients [16,43,44]. We found a strong association between TIM-3 and CD96 in CD3^+^ (CD4^+^ and CD8^+^) T cells. CD96 is a newly proposed target for IC inhibitors in solid cancers, and little is known about its role in AML [45]. Based on its prognostic significance, the co-inhibition of TIM-3 and CD96 may be promising and deserves further research. We also observed a significant association between TIM-3 and PD-1 in CD3^+^ (and CD8^+^) T cells. A previous study showed an increased frequency of PD-1^+^TIM-3^+^CD4^+^ and PD-1^+^TIM-3^+^CD8^+^ T cells in the BM of ND-AML patients, but did not establish whether it has prognostic significance [27]. Our data, which included more ND-AML cases, showed the prognostic significance of PD-1^+^TIM-3^+^CD3^+^ (CD8^+^) T cells as well as their increased frequency. This double-positive cell population has been associated with immune exhaustion in animal models of AML [46] and relapse after all HSCTs in patients with AML [47]. Given that TIM-3 has also been restricted to surface leukemic stem cells in AML [43], the current study points to TIM-3 as an ideal candidate for T cell-harnessing immunotherapies. Several phase I/II trials with an anti-TIM-3 antibody as a monotherapy or in combination with various drugs are currently underway [43]. We strongly suggest that co-inhibition of PD-1 and TIM-3 may improve its efficacy, which should be tested in future trials.

Despite increasing interest in γδ T cells as an immunotherapeutic platform, little is known about their distribution, characteristics, and subtypes in chemotherapy settings in AML [1,22,48]. We observed that γδ T cells were present in the BM at varying frequencies without differences between the ND-AML and HC groups, which did not change after chemotherapy. The current study did not find an association between γδ T cell frequency and clinical outcomes, contradicting previous transcriptomic data linking tumor-infiltrating Vδ2 γδ T cells to better survival in patients with AML [48]. Interestingly, the IC receptor expression patterns differ between Vδ1 and Vδ2 γδ T cells. The Vδ1 subtypes had more γδ T cells expressing inhibitory IC receptors (PD-1, TIGIT, and CD112R), while the Vδ2 subtypes had more activating IC receptors (DNAM-1 and OX40). Similar findings in the HCs suggest that the Vδ2 γδ T cells may have stronger anti-leukemic properties than the Vδ1 γδ T cells in the BM. This may indicate the potential benefit of the Vδ2 subtypes as an immunotherapeutic platform, such as CAR-γδ T cells. Meanwhile, the more prevalent expression of activating IC receptors (NKG2D and NKp30) on the Vδ2 than Vδ1 subtypes in the HCs was reversed in the ND-AML patients, which may be related to the failure of immune surveillance by γδ T cells. The Vδ2 γδ T cells in the ND-AML patients showed decreased NKG2D and NKp30 expression compared to those of the HCs. Additionally, the γδ T cells were exhausted mainly by an increase in TIGIT^+^ cells, similar to conventional T cells. Effective chemotherapy led to a partial recovery of exhausted γδ T cells, as indicated by the decrease in TIGIT^+^ and/or TIM-3^+^ Vδ1 and Vδ2 subtypes at the CR stage. Meanwhile, we also observed a reduced frequency of NKG2D^+^ γδ T cells in patients at the CR stage and the R/R stage compared to the ND-AML stage, suggesting that chemotherapy may be involved. These changes in IC proteins on γδ T cells may determine the anti-leukemic properties at the CR stage, which is supported by our data showing a correlation between TIGIT^+^Vδ1 subtypes and the inverse correlation of OX40^+^Vδ2 subtypes with *WT1* levels, which was not observed in conventional T cells. This suggests that γδ T cells may be crucial in eliminating the remaining leukemic cells in remission. Meanwhile, as in conventional T cells, we revealed the prognostic importance of TIM-3^+^Vδ2 T cells and their association with CD96^+^ cells and observed a significant association between PD-1 and TIM-3 in the Vδ1 and Vδ2 subtypes. The proportion of PD-1^+^TIM-3^+^ γδ T cells was higher than that of conventional T cells, which negatively affected survival outcomes both in the Vδ1 and Vδ2 subtypes. DNAM-1^−^TIGIT^+^CD96^+^ T cells and NK cells in the PB are associated with poor survival in AML [49]. Our results revealed that DNAM-1^−^TIGIT^+^CD96^+^ expression in γδ T cells was predictive of a lower survival rate. Collectively, the current study suggests that exhausted γδ T cells may be important in immune escape, particularly at the CR stage, and the prognosis of patients with AML based on the phenotype, but not the number, of γδ T cells.

A variety of IC ligands on blasts for each receptor were also evaluated in this study. We found that IC ligand expression varied, but there was no difference between the diagnosis and R/R stages, and it did not have any prognostic value. However, we cannot conclusively determine the prognostic relevance of each ligand and/or receptor because our data only represent a snapshot of time for these patients. Moreover, much uncertainty remains about the precise mechanisms and outcomes of the interactions between IC receptors and their corresponding ligands. In this context, we showed the potential of multiple regression modeling that incorporates diverse patterns of receptors and ligands in each patient that we used to predict survival outcomes, reinforcing previous transcriptomic data that stratified the BM microenvironment into immune subtypes [26]. This suggests that immunological stratification based on the phenotypic features of IC receptors and ligands in pretreated BM samples, in conjunction with cytogenetic and mutational information, may enable risk prediction.

## 4. Materials and Methods

### 4.1. Sample Collection

BM samples from patients with AML were obtained as part of an acute leukemia cohort study, which was registered with the Clinical Research Information Service (#KCT0002261), and BM samples at the time of diagnosis, remission, and R/R after chemotherapy were also longitudinally collected. This study included 13 healthy controls (HCs) and 89 patients with non-promyelocytic AML, of whom, 71 were newly diagnosed with AML (ND-AML) and 40 were in the R/R stage. BM samples from 47 patients who achieved complete remission (CR) according to the European LeukemiaNet (ELN) 2022 criteria after intensive chemotherapy were also included. Paired samples at the stages of ND-AML and CR, CR and relapse, or ND-AML and R/R were collected from 38, 20, and 26 patients, respectively. Supplementary Table S5 describes the details of all the enrolled patients. There were no significant differences between the ND-AML and HC groups in terms of age or sex (Supplementary Figure S11). All patients received intensive induction chemotherapy with 3 days of anthracycline and 7 days of cytarabine. Fresh BM samples were collected using sterile heparinized syringes. The cells were then centrifuged using Ficoll-Paque (GE Healthcare, Seoul, Republic of Korea) to separate the buffy coats based on the Ficoll gradient density. The buffy coat was immediately used for flow cytometry analysis or was preserved in liquid nitrogen and after one day, it was stored in a −80 °C deep freezer for future use. Fresh BM samples were collected using sterile heparinized syringes. Bone marrow mononuclear cells (BM-MNCs) were isolated by layering the bone marrow sample onto Ficoll-Paque (GE Healthcare) and centrifuging at 2500 rpm for 20 min at room temperature. The BM-MNCs were carefully extracted from the buffy coat at the interphase. The isolated BM-MNCs were either immediately used for flow cytometry analysis or cryopreserved in a freezing medium containing 10% dimethyl sulfoxide (DMSO) and 90% fetal bovine serum (FBS). BM-MNCs were first stored at −80 °C for 24 h and then transferred to liquid nitrogen for long-term preservation to maintain high viability.

### 4.2. Thawing Protocol

Among the 158 AML samples (at diagnosis, N = 71; at remission, N = 47; at relapsed/refractory, N = 40) and 13 HC samples, freshly isolated buffy coat (AML, N = 111; HC, N = 13) and thawed samples (AML, N = 47) were used for flow cytometry analysis. When we thawed a cryovial, the BM-MNCs were incubated for 30 min at 37 °C in a CO_2_ incubator. They were cultured in complete Alpha MEM (Welgene, Daegu, South Korea) supplemented with 10% fetal bovine serum (FBS, Youngin Frontier, Seoul, South Korea) and 1% Penicillin/Streptomycin (Gibco Laboratories, Grand Island, NY, USA). To prevent aggregation when using thawed samples, 200 mM MgSO_4_ (Sigma-Aldrich, St. Louis, MO, USA), 2 mg/mL heparin (Alfa Aesar, Ward Hill, MA, USA), and 1 mg/mL DNase (Sigma-Aldrich, St. Louis, MO, USA) were added. These additives also help digest the DNA from dead cells and prevent coagulation. The expression of various target proteins was analyzed using a Cytek Aurora™ flow cytometer (Cytek Biosciences, Fremont, CA, USA).

### 4.3. Multiparameter Flow Cytometry

The bone marrow mononuclear cells from AML patients were examined using a Cytek Aurora™ flow cytometer (Cytek Biosciences, Fremont, CA, USA) and T cell immunophenotyping was performed. The cells were harvested and stained with antibodies targeting proteins associated with intracellular components (IC proteins) and appropriate isotype-matched antibodies were used as negative controls. Approximately 1,000,000 live bone marrow cells were analyzed per sample. The cells were washed with FACS buffer (PBS (Welgene) with 2% FBS). Surface staining was performed by adding surface antibodies to the cell suspension in 100 μL of FACS buffer. After 30 min of incubation, the cells were washed with FACS buffer. The expression of various target proteins was analyzed using a Cytek Aurora™ flow cytometer (Cytek Biosciences, Fremont, CA, USA). The acquired data were compensated for using SpectroFlo v3.0 software (Cytek Biosciences, Fremont, CA, USA) and analyzed using FlowJo v10 software (BD Biosciences, Franklin Lakes, NJ, USA).

Nine panels with different antibodies to gate and stain IC receptors and ligands were used (Supplementary Methods S1). The presence of lymphocyte subsets was analyzed to obtain an overview of their general distribution in the BM (panel 1). CD45RO and CCR7 were used to distinguish between the maturation and memory states of T cells (panel 9). We evaluated clinically relevant inhibitory and activated IC receptors in the following T cell subsets: CD3^+^, CD4^+^, CD8^+^, and γδ T cells (Vδ1 and Vδ2 subtypes) (panels 2~4). The AML blasts were assessed for the expression of the corresponding IC ligands (panels 5~8). The gating strategies (Supplementary Methods S2) and other detailed materials regarding the experiments are shown in the Supplementary Methods S3.

### 4.4. Statistical Analysis

The data are presented as the median, range, frequency, and percentage. Continuous variables were compared using the Mann–Whitney U test and categorical variables were compared using the chi-square or Fisher’s exact test. To determine the correlation between two continuous variables, the Spearman’s rank correlation coefficient (ρ) was calculated and the *p*-value was obtained using the confidence interval. Survival outcomes were compared using Kaplan–Meier estimates and log-rank tests. Kendall’s tau test was used to perform trend analysis. For the multivariate survival analysis, we built Cox proportional hazards models using IC receptor expression and clinical parameters as variables. Additionally, we performed modeling to predict survival using IC receptor expression in T cells and IC ligand expression in AML blasts as variables; L1 penalized regression (Lasso regularization) was used and the λ that maximizes the model’s Harrell’s C-index was estimated through 5-fold cross-validation. R Statistical Software (v4.3.1; R Core Team 2023) was used to perform all the analyses and to generate the figures. Statistical significance was indicated by * *p* < 0.05, ** *p* < 0.01, *** *p* < 0.001, and **** *p* < 0.0001.

## 5. Conclusions

The novelty of this study is the large number of longitudinal samples and the number of markers investigated in the analyses involving blasts and T cell subsets. In addition, this study is the first to examine γδ T cells for the distribution and expression of IC receptors in the BM of AML patients, revealing the prognostic value of exhausted patterns in γδ T cells not only at diagnosis but also at remission. Our data provide additional evidence for the heterogeneity of the BM immune environment and the immunological stratification of BM samples, highlighting the phenotype, but not the number, of T cell subsets. Furthermore, the current comprehensive phenotype analysis of both T cell subsets and blasts not only revealed the importance of TIGIT, TIM-3, CD96, PD-1^+^TIM-3^+^, and DNAM-1-TIGIT^+^CD96^+^ cells, but it also showed the potential of risk modeling using various patterns of IC receptors and their ligands. Our findings need to be validated in independent cohorts and other treatment settings. We acknowledge that our study did not cover all the elements of immune biology, including functional aspects of T cell subsets and other possible contributors and components of the BM microenvironment. However, our data can help us understand the T cell and IC landscape in AML, which can spur further research and help identify the optimal targets and conditions for T cell-harnessing immunotherapies.

## Data Availability

For the original data, please contact to corresponding author (cbscho@catholic.ac.kr).

## Supplementary Materials

The following supporting information can be downloaded at: https://www.mdpi.com/article/10.3390/ijms252313039/s1.
