# Supplementary material for: Characterization of the Bone Marrow Lymphoid Microenvironment and Discovery of Prognostic Immune-Related Factors in Acute Myeloid Leukemia
Source: Int J Mol Sci. 2024 Dec 4;25(23):13039. doi: 10.3390/ijms252313039 (PMC11641137; doi:10.3390/ijms252313039)
Supplement: Supplementary file 1 [file ijms-25-13039-s001.zip › Supplementary Figures - IJMS - Revised.pdf]

Supplementary Figure S1. Changes in phenotype percentage with age in each T-cell subset of patients with newly diagnosed AML. Within the T cell subsets, the phenotypes of EM, CM, TE, and Naïve T cells were color-coded and the proportion of expression of each phenotype was pointed according to the age of each patient. A linear trend line was plotted for each phenotype. Asterisks indicate the significance of Spearman's rank correlation coefficient. $CD8^+$ were defined as $CD4^-$ T cells among $CD3^+TCR\alpha\beta^+$ cells. ND: Newly diagnosed; AML: Acute myeloid leukemia; TE: effector T-cell; CM: Central memory T-cell; EM: Effector memory T-cell

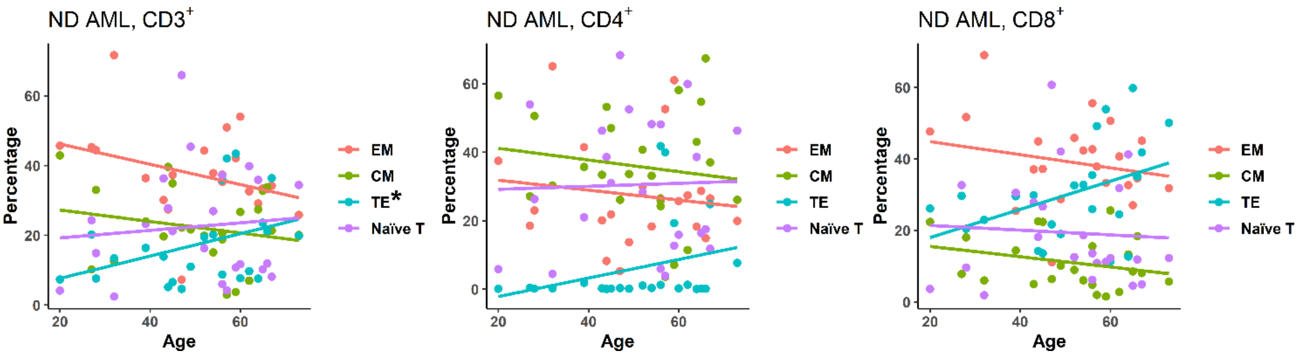

Supplementary Figure S2. BM T-cell IC receptor expression in pooled samples of relapsed, and refractory AML patients (N: Rel = 23; Ref = 17). The p-values for the pooled sample data are calculated using the Mann–Whitney U test. CD8$^+$ were defined as CD4$^-$ T cells among CD3$^+$TCRαβ$^+$ cells. AML, acute myeloid leukemia; BM, bone marrow; IC, immune checkpoint; Rel, relapse; Ref, refractory.

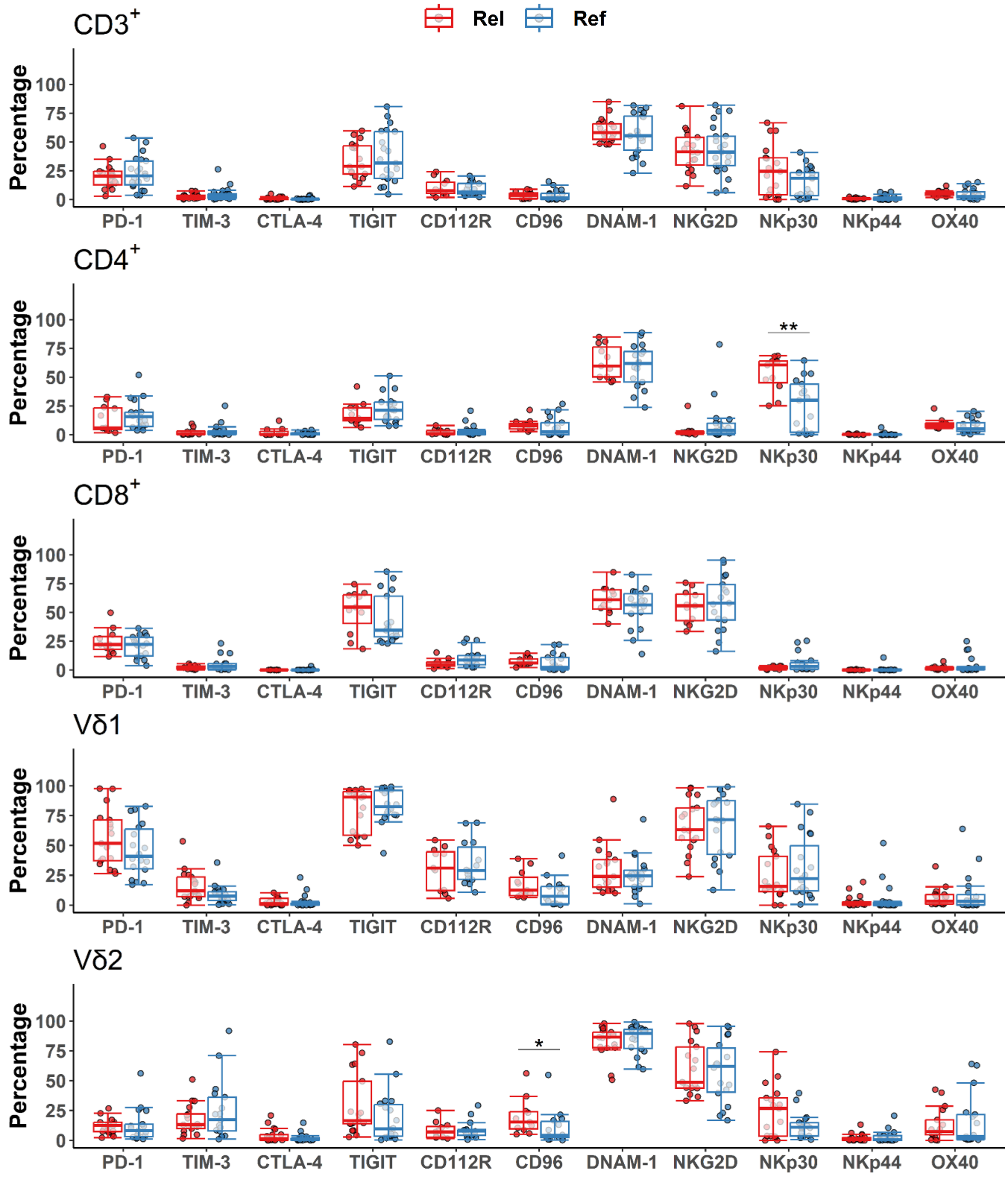

Supplementary Figure S3. Changes in T-cell subset frequency according to disease state. The expression of each lymphocyte subset in the all available patients was examined and compared whether they differed by disease state. Asterisks indicate statistically significant. CR: Complete remission; Lym: Lymphocyte; ND: Newly diagnosed; NS: non-significant; R/R: relapsed/Refractory. p-values of < 0.0001 were marked as “****”, ≥ 0.001 and < 0.01 as “**”, and ≥ 0.01 and < 0.05 as “*”.

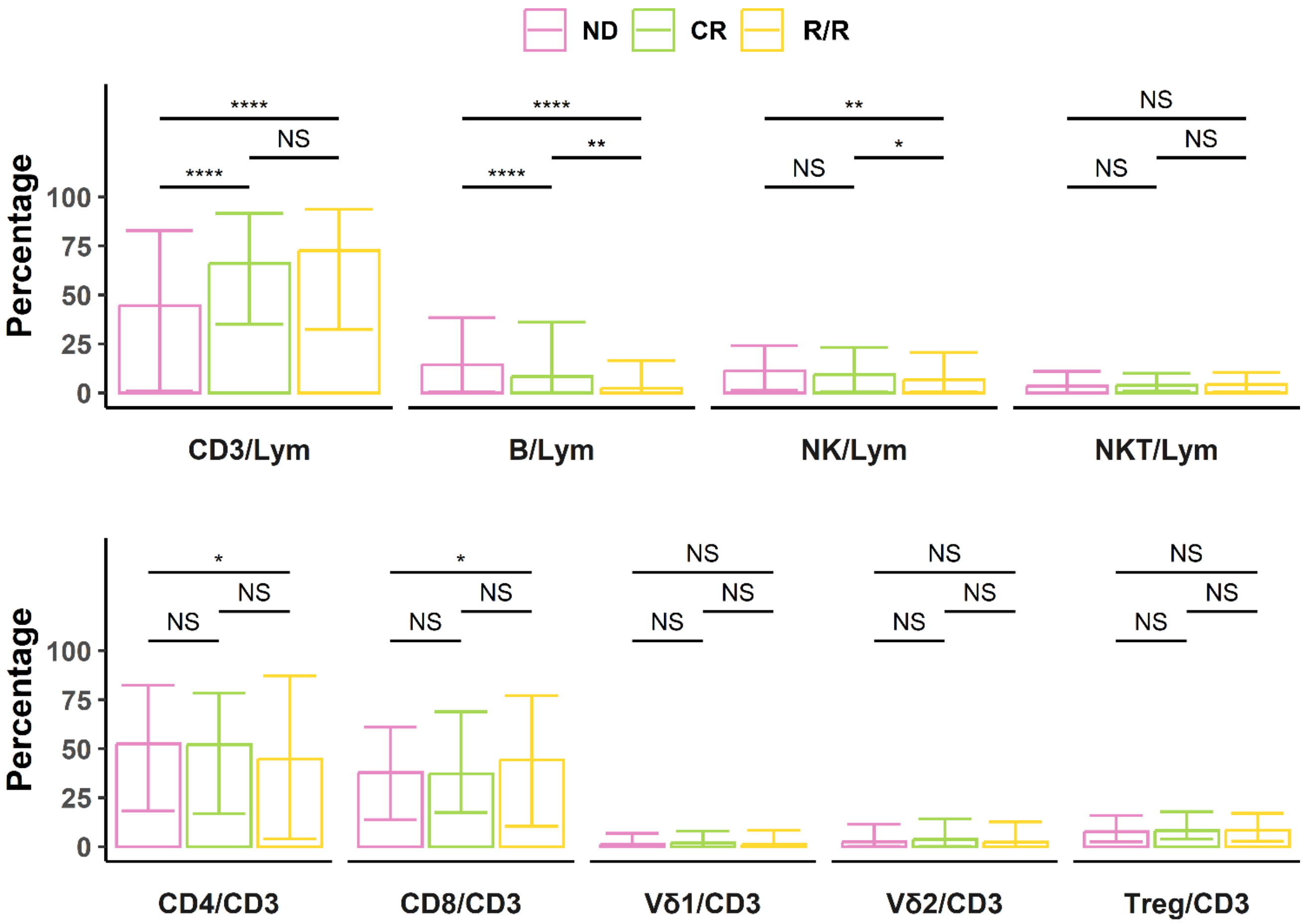

Supplementary Figure S4. Changes in T-cell phenotype according to disease state. The expression of each T cell phenotye in the all available patients was examined and compared whether they differed by disease state. Asterisks indicate statistically significant. CM: Central memory T-cell; CR: Complete remission; EM: Effector memory T-cell; ND: Newly diagnosed; NS: Non-significant; R/R: Relapsed/Refractory; TE: Effecter T-cell. p-values of ≥ 0.001 and < 0.01 as “**”, and ≥ 0.01 and < 0.05 as “*”.

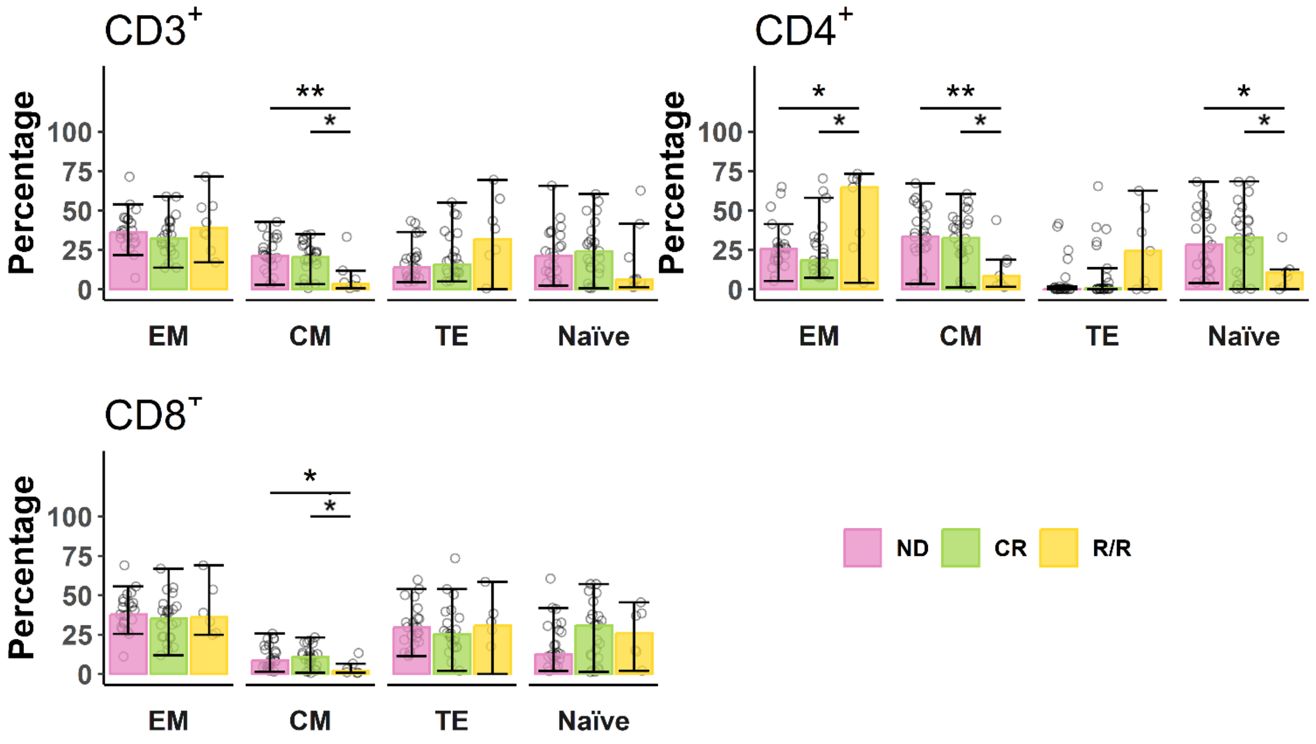

Supplementary Figure S5. Overall survival of patients from relapse according to TIGIT change status at relapse. 'Increased' groups are those with increased TIGIT expression compared to remission at the time of relapse, and 'Decreased' groups are those with decreased or no increase in TIGIT expression compared to remission at the time of relapse. The p-value in the survival curve is based on the log-rank test. OS, overall survival.

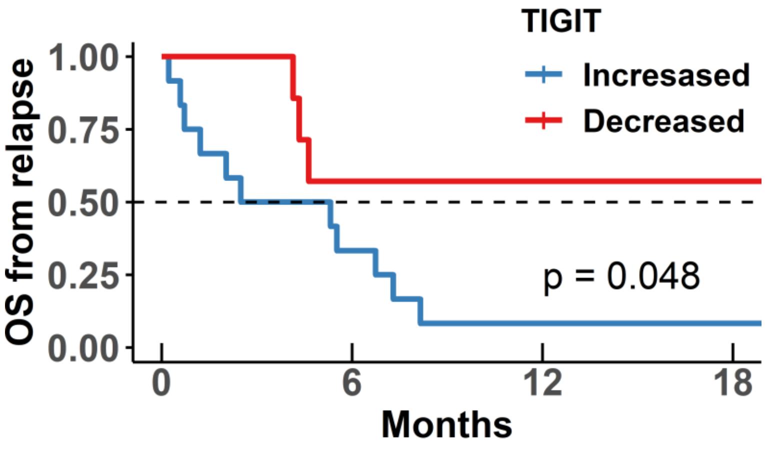

Supplementary Figure S6. Comparison of IC receptor expression between BM Vδ1 and Vδ2 ɣδT-cells in healthy controls. BM: Bone marrow; HC: Healthy controls; IC: Immune checkpoint. NS: non-significant. “****”, ≥ 0.001 and < 0.01 as “**”, and ≥ 0.01 and < 0.05 as “*”.

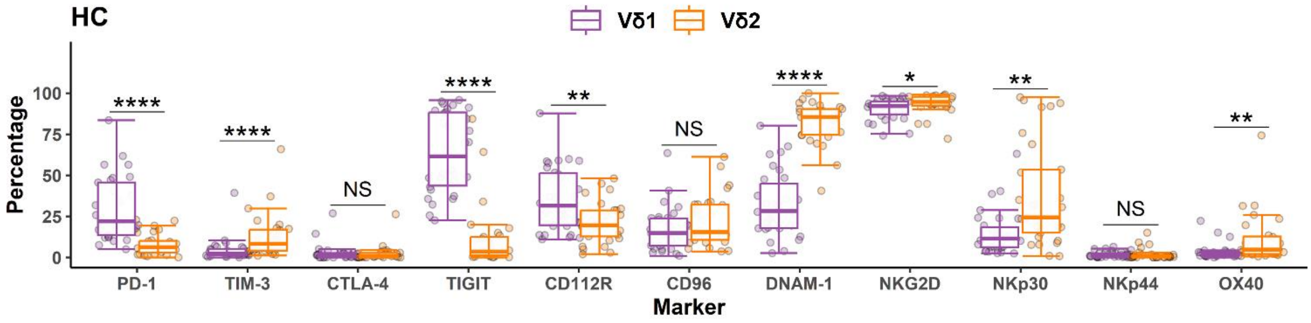

Supplementary Figure S7. Correlations among immune checkpoint receptor expression in Vδ1 and Vδ2 γδT-cells. Numbers and marks indicate Spearman's rank correlation coefficient and their significance, respectively. p-values of ≥ 0.0001 and < 0.001 as "***", ≥ 0.001 and < 0.01 as "**", and ≥ 0.01 and < 0.05 as "*".

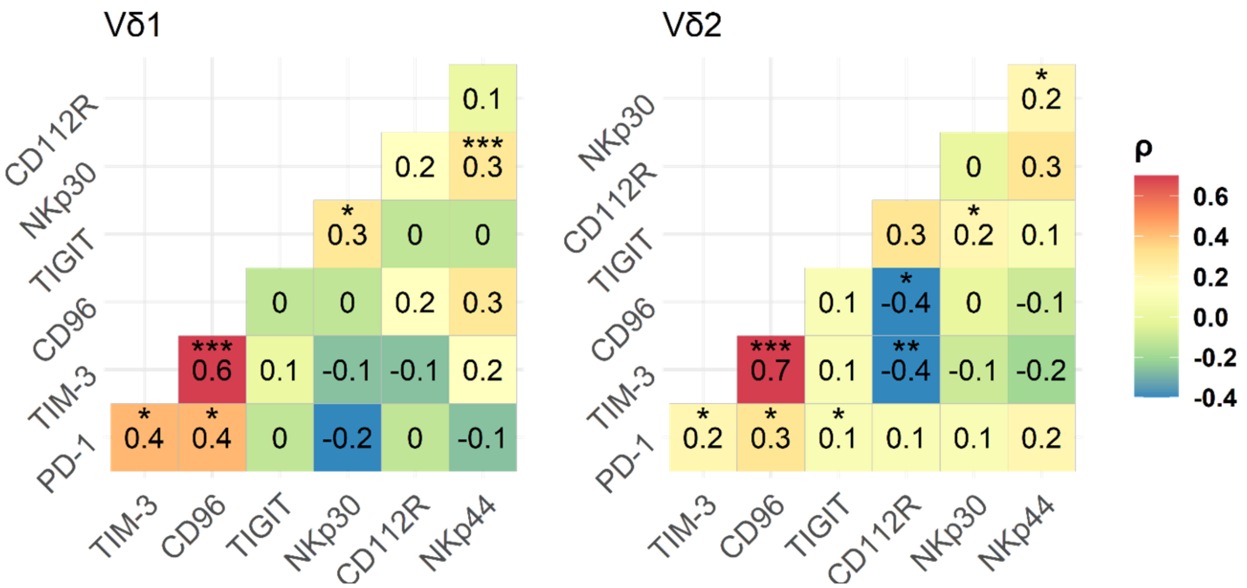

Supplementary Figure S8. IC ligand expression in leukemic blasts. The ligand expression of immune checkpoint receptors in AML blast cells from all available patients was examined and compared between ND and R/R state. AML: Acute myeloid leukemia; ND: Newly diagnosed; R/R: Relapsed/Refractory. P-values were from Mann Whitney U tests.

0

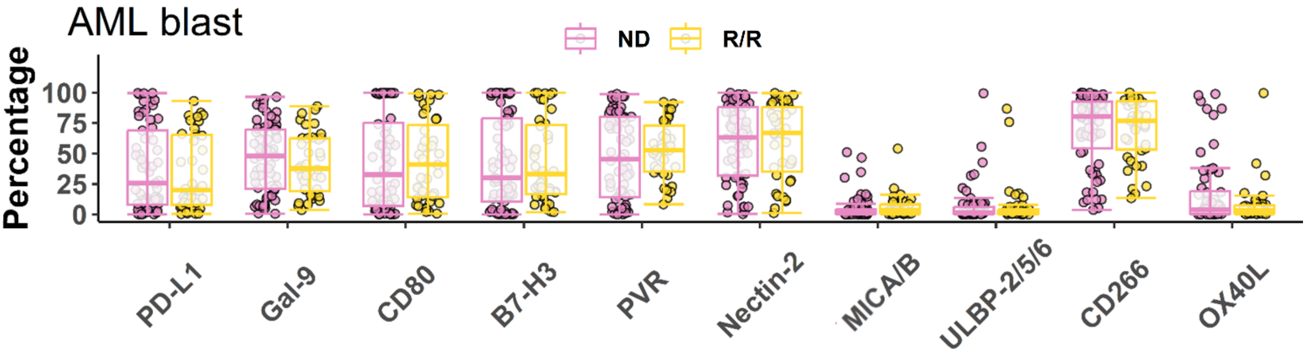

Supplementary Figure S9. Comparison of PD-1⁺TIM-3⁺ and DNAM-1⁻TIGIT⁺CD96⁺ T-cell proportions among T-cell subsets. The proportion of gating positive cells within T-cell subset of each patients was examined and compared for significant differences in expression between T-cell subsets. P values were calculated by Mann-Whitney test. $CD8^+$ T cells were defined as $CD4^-$ T cells among $CD3^+TCR\alpha\beta^+$ cells. AML: Acute myeloid leukemia, ND: Newly diagnosed.

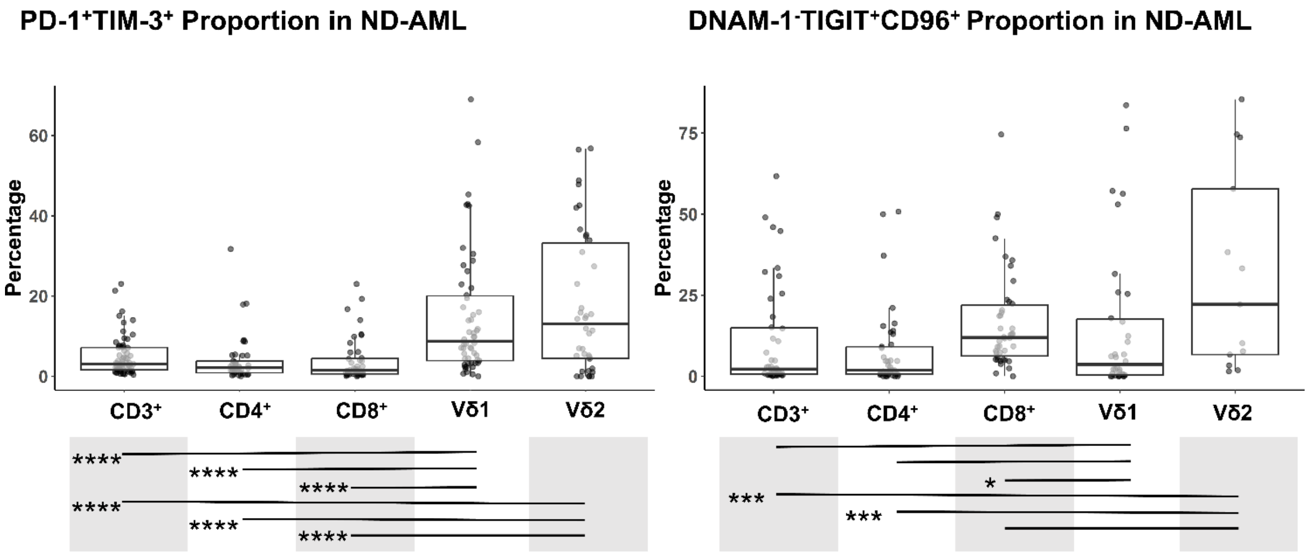

Supplementary Figure S10. Changes in coefficients of immune checkpoint receptor variables with λ in penalized (L1 regularization) survival models with variables. How the coefficient (β) of each variable varies depending on the λ value in L1 regularization model was plotted. Variables with present from low λ with high β values can be considered to be highly significant.

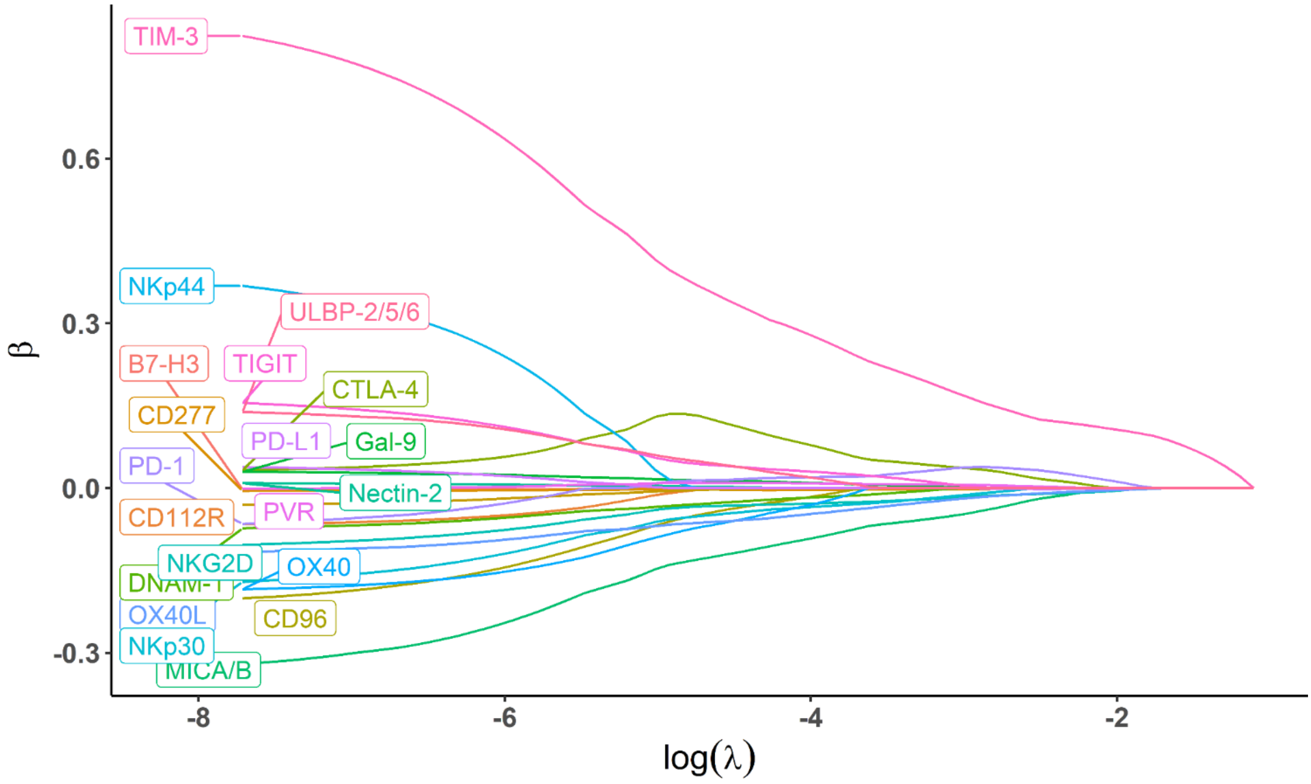

Supplementary Figure S11. Comparison of age and sex between healthy controls and patients with newly diagnosed AML. A. Age distribution of HC and ND-AML. The P-value was calculated using the Mann-Whitney U test. **B.** Sex distribution of HC and ND-AML. The P-values were calculated using the Fisher's exact test. AML: Acute myeloid leukemia; HC: Healthy controls; ND: Newly diagnosed.

A.

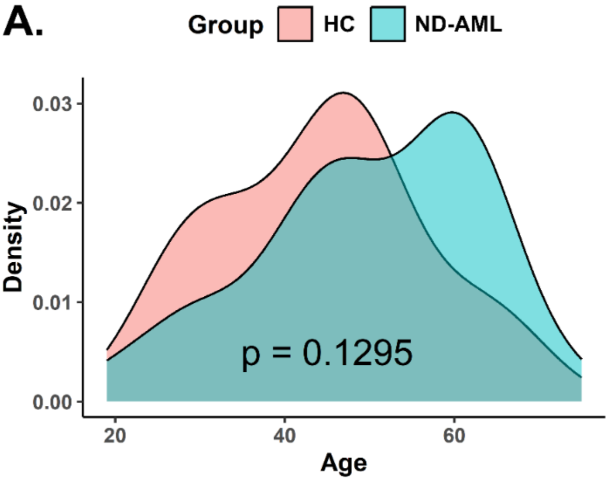

B.

N, %	HC (N: 13)	ND-AML (N: 71)	p
**Female**	5 (38.5)	23 (32.4)	1.0
**Male**	8 (61.5)	44 (62.0)	
