# Supplementary material for: Characterization of the Bone Marrow Lymphoid Microenvironment and Discovery of Prognostic Immune-Related Factors in Acute Myeloid Leukemia
Source: Int J Mol Sci. 2024 Dec 4;25(23):13039. doi: 10.3390/ijms252313039 (PMC11641137; doi:10.3390/ijms252313039)
Supplement: Supplementary file 1 [file ijms-25-13039-s001.zip › Supplementary Methods - IJMS - Revised.pdf]

Supplementary Methods S1. Gating panel fluorescence, marker, clone, and company

Nine panels with different antibodies to gate and stain IC receptors and ligands were used (Supplementary Methods S1). The presence of lymphocyte subsets was analyzed to obtain an overview of their general distribution in BM (panel 1). CD45RO and CCR7 were used to distinguish between the maturation and memory states of T cells (panel 9). We evaluated clinically relevant inhibitory and activated IC receptors in the following T cell subsets: CD3+, CD4+, CD8+, and $\gamma\delta$ T cells ($V\delta1$ and $V\delta2$ subtypes)(panels 2~4). The AML blasts were assessed for their corresponding IC ligands (panels 5~8).

- ✓ Panels 1~9 for samples at diagnosis or relapsed/refractory:
- ✓ Panels 1, 2, 3, 4, and 9 for samples at remission or healthy control

Panel 1	Flourscence	FITC	PE-610	PerCP-Cy5.5	PE-Cy7	APC	APC-Cy7	BV421	BV510	BV605
	Marker	CD3	CD56	CD19	CD127	CD25	CD8	CD4	Live dead	CD45
	Clone	UCHT1	CMSSB	HIB19	eBioRDR5	BC96	RPA-T8	OKT4 (OKT-4)	-	2D1
	Source/Company	Invitrogen	Invitrogen	Invitrogen	Invitrogen	Invitrogen	Invitrogen	Invitrogen	Invitrogen	Invitrogen

Panel 2	Flourscence	FITC	PE	PE-610	PerCP-Cy5.5	PE-Cy7	APC	Alexa Flour700	APC Cy7	BV421	BV510	BV605	BV711
	Marker	CD3	CD112R	CD96(Tactile)	NKp44	TCRVd1	TIGIT	CD56	NKG2D	DNAM1	Live dead	TCRVd2	CD4
	Clone	UCHT1	W16216D	NK92.39	44.189	TS8.2	MBSA43	HCD56	1D11	11A8	-	B6	RPA-T4
	Source/Company	Invitrogen	Biolegend	Biolegend	Invitrogen	Invitrogen	Invitrogen	Biolegend	Biolegend	Biolegend	Invitrogen	Biolegend	Biolegend
Iso type (control)	Marker		IgG 1,k	IgG 1,k	IgG 2b,k		IgG 1,k		IgG 1,k	IgG 1,k			
	Clone		MOPC-21	G043H7	eBMG2b		P3.6.2.8.1		MOPC-21	X40			
	Source/Company		BD	Biolegend	Invitrogen		Invitrogen		Biolegend	BD			

Panel 3	Flourscence	FITC	PE	PE-610	PE-Cy7	APC	Alexa Flour700	BV421	BV510	BV605	BV711
	Marker	CD3	PD-1	TIM3	TCRVd1	CD1d tet	CD56	CTLA4	Live dead	TCRVd2	CD4
	Clone	UCHT1	eBioJ105 (J105)	F38-2E2	TS8.2	Home made	HCD56	BNI3	-	B6	RPA-T4
	Source/Company	Invitrogen	Invitrogen	Invitrogen	Invitrogen	NIH Tetramer Core Facility	Biolegend	Biolegend	Invitrogen	Biolegend	Biolegend
Iso type (control)	Marker		IgG 1,k	IgG 1,k				IgG 2a,k			
	Clone		MOPC-21	P3.6.2.8.1				G155-178			
	Source/Company		BD	Invitrogen				BD			

Panel 4	Flourscence	FITC	PE	PE-610	PE-Cy7	APC	Alexa Flour700	BV510	BV605	BV711
	Marker	CD3	NKp30	OX40	TCRVd1	CXCR4	CD56	Live dead	TCRVd2	CD4
	Clone	UCHT1	AF29-4D12	Ber-ACT35 (ACT35)	TS8.2	12G5	HCD56	-	B6	RPA-T4
	Source/Company	Invitrogen	Invitrogen	Biolegend	Invitrogen	Invitrogen	Biolegend	Invitrogen	Biolegend	Biolegend
Iso type (control)	Marker		IgG 1,k	IgG 1,k		IgG 2a,k				
	Clone		MOPC-21	G043H7		G155-178				
	Source/Company		BD	Biolegend		BD				

**Panel 5**	**Flourscence**	**FITC**	**PE**	**PerCP-Cy5.5**	**APC**	**APC-Cy7**	**BV421**	**BV510**	**BV605**
	**Marker**	**CD38**	**Nectin-2**	**MICA/B**	**ULBP-2/5/6**	**PVR**	**CD34**	**Live dead**	**CD33**
	**Clone**	**HIT2**	**TX31**	**6D4**	**165903**	**SKII.4**	**4H11**	**-**	**WM-53 (WM53)**
	**Source/Company**	**Invitrogen**	**Biolegend**	**Biolegend**	**R&D systems**	**Biolegend**	**Invitrogen**	**Invitrogen**	**Invitrogen**
**Iso type (control)**	**Marker**		**IgG 1,k**	**IgG 2a,k**	**IgG 2a**	**IgG 1,k**			
	**Clone**		**MOPC-21**	**MOPC-173**	**-**	**MOPC-21**			
	**Source/Company**		**BD**	**Biolegend**	**Bio-Techne**	**Biolegend**			

**Panel 6**	**Flourscence**	**FITC**	**PE**	**PerCP-Cy5.5**	**PE-Cy7**	**APC**	**BV421**	**BV510**	**BV605**
	**Marker**	**CD38**	**PD-L1**	**CD80**	**CD34**	**Galectin9**	**OX40L**	**Live dead**	**CD33**
	**Clone**	**HIT2**	**MIH1**	**2D10.4**	**4H11**	**9M1-3**	**ik-1**	**-**	**WM-53 (WM53)**
	**Source/Company**	**Invitrogen**	**BD**	**Invitrogen**	**Invitrogen**	**Invitrogen**	**BD**	**Invitrogen**	**Invitrogen**
**Iso type (control)**	**Marker**		**IgG 1,k**	**IgG 1,k**		**IgG 1,k**	**IgG 2a,k**		
	**Clone**		**MOPC-21**	**P3.6.2.8.1**		**P3.6.2.8.1**	**G155-178**		
	**Source/Company**		**BD**	**Invitrogen**		**Invitrogen**	**BD**		

**Panel 7**	**Flourscence**	**FITC**	**PE**	**PerCP-Cy5.5**	**PE-Cy7**	**BV510**	**BV605**
	**Marker**	**CD38**	**CD277**	**B7-H3**	**CD34**	**Live dead**	**CD33**
	**Clone**	**HIT2**	**232-5**	**7-517**	**4H11**	**-**	**WM-53 (WM53)**
	**Source/Company**	**Invitrogen**	**BD**	**Invitrogen**	**Invitrogen**	**Invitrogen**	**Invitrogen**
**Iso type (control)**	**Marker**		**IgG 2a,k**	**IgG 1,k**			
	**Clone**		**G155-178**	**P3.6.2.8.1**			
	**Source/Company**		**BD**	**Invitrogen**			

**Panel 8**	**Flourscence**	**FITC**	**PerCP-Cy5.5**	**PE-Cy7**	**BV510**	**BV605**
	**Marker**	**CD38**	**CD1d**	**CD34**	**Live dead**	**CD33**
	**Clone**	**HIT2**	**51.1**	**4H11**	**-**	**WM-53 (WM53)**
	**Source/Company**	**Invitrogen**	**Invitrogen**	**Invitrogen**	**Invitrogen**	**Invitrogen**
**Iso type (control)**	**Marker**		**IgG 2b,k**			
	**Clone**		**eBMG2b**			
	**Source/Company**		**Invitrogen**			

**Panel 9**	**Flourscence**	**FITC**	**PerCP-Cy5.5**	**APC**	**APC-Cy7**	**BV421**	**BV510**
	**Marker**	**CD3**	**CCR7**	**CD8**	**CD45RO**	**CD4**	**Live dead**
	**Clone**	**UCHT1**	**G043H7**	**OKT8(OKT-8)**	**UCHL1**	**OKT4 (OKT-4)**	**-**
	**Source/Company**	**Invitrogen**	**Biolegend**	**Invitrogen**	**BD**	**Invitrogen**	**Invitrogen**

Supplementary Methods S2. Gating strategies. AML: Acute myeloid leukemia; BM: Bone marrow; HC: Healthy control; ND: Newly diagnosed.

Panel 1: frequency of the immune subset for healthy control

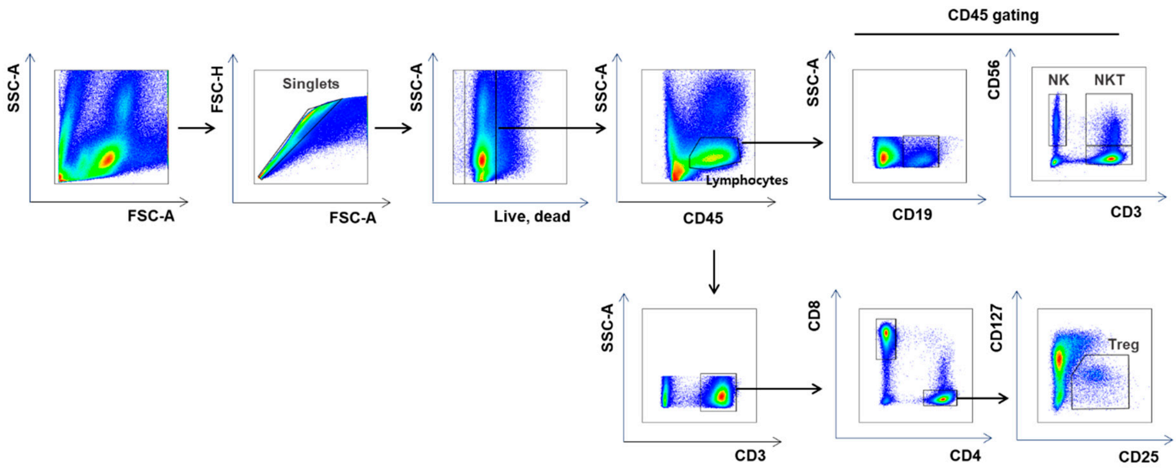

Panel 1 was used to detect and gate the presence of lymphocyte subsets. The above figure is an example of BM samples from healthy controls.

Panels 2, 3, and 4: immune checkpoint receptors for healthy control

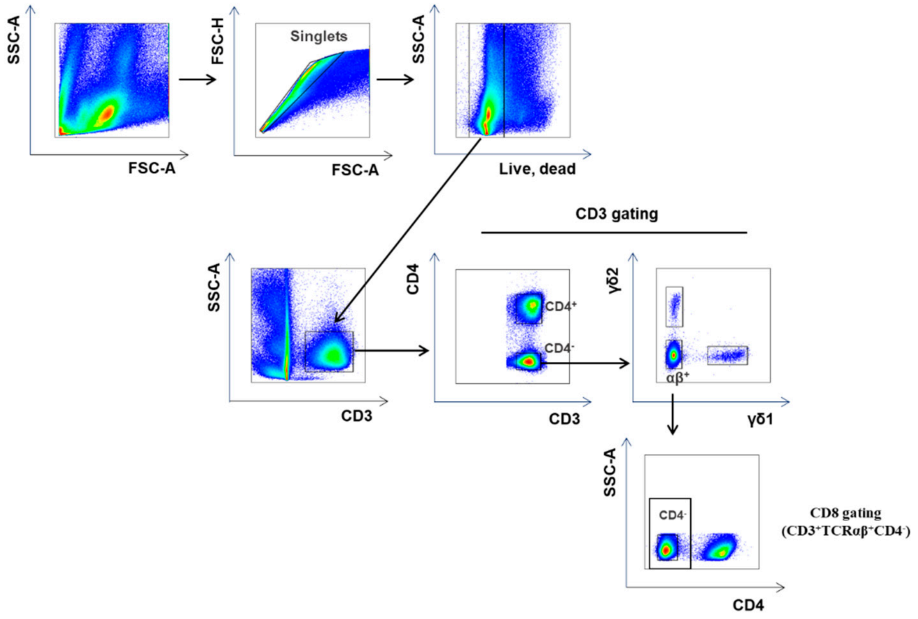

Panels 2, 3, and 4 were used to gate lymphocyte subsets for analysis of inhibitory and activated IC receptors. The above figure is an example of BM samples from healthy controls.

Panel 1: frequency of the immune subset for AML patients

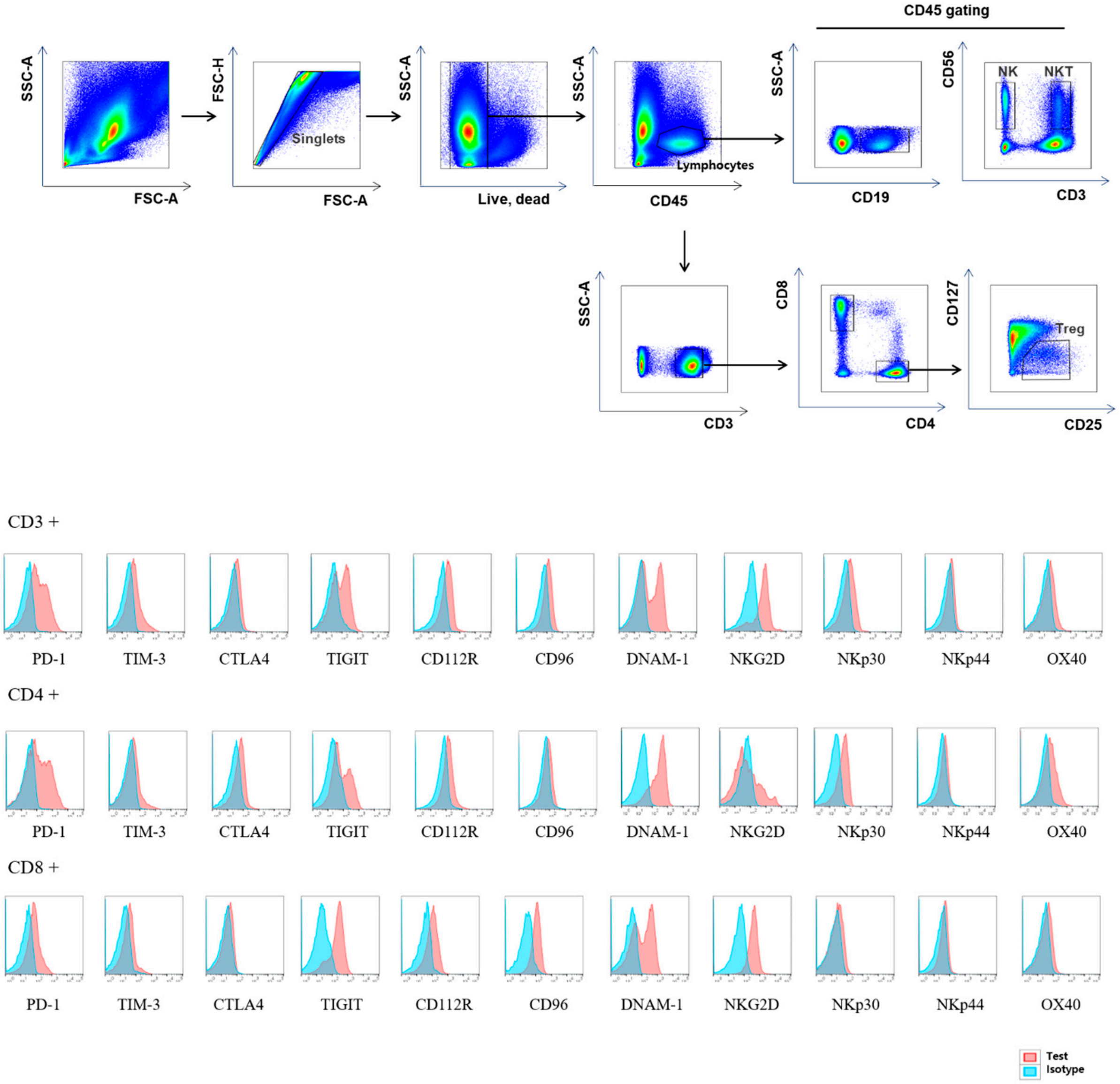

Panel 1 was used to detect and gate the presence of lymphocyte subsets. The above figures are examples of BM samples from patients with AML. The lower panel shows histograms for each IC receptor on each immune subset.

Panels 2, 3, and 4: immune checkpoint receptors for AML patients

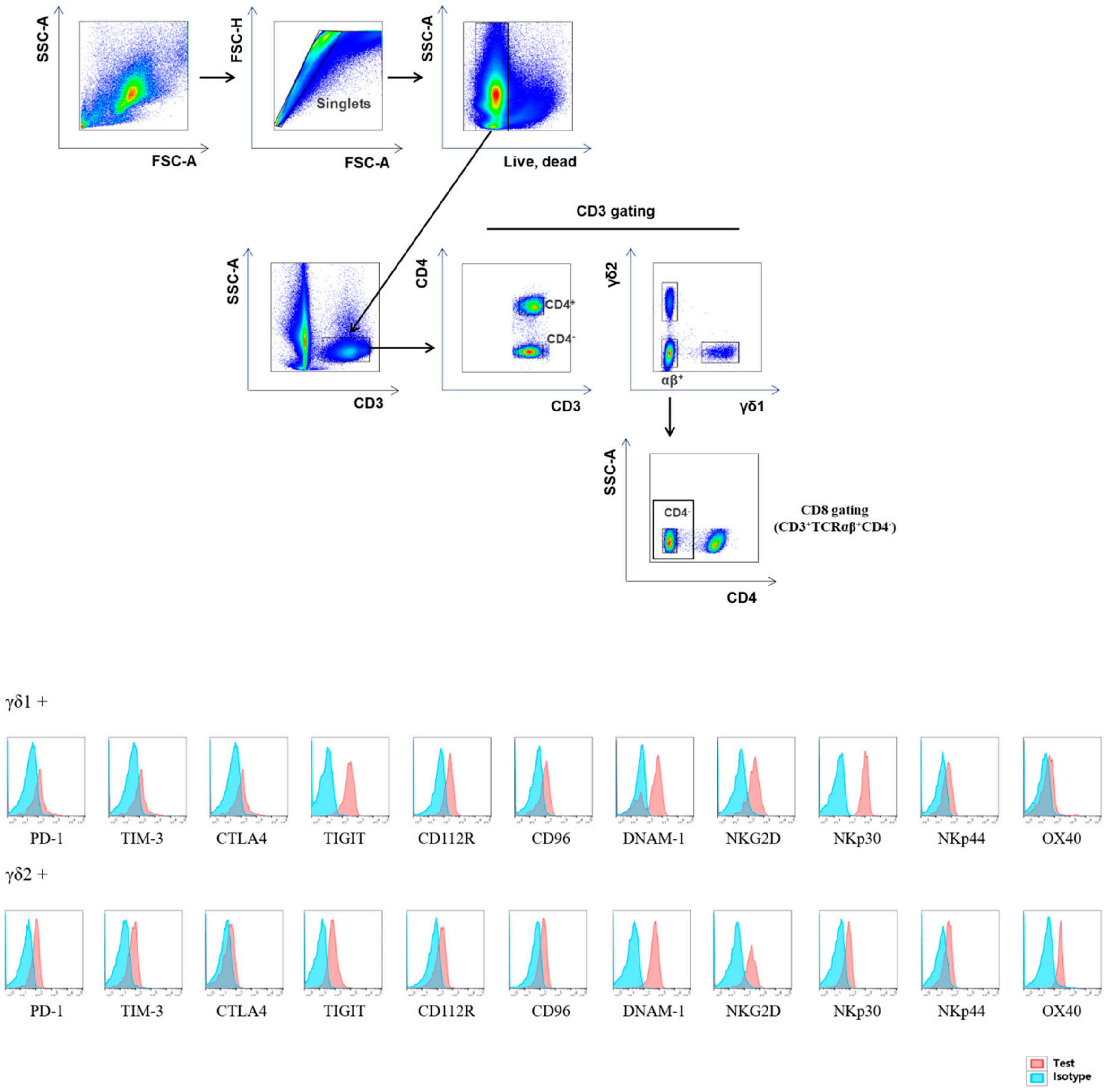

Panels 2, 3, and 4 were used to gate lymphocyte subsets for analysis of inhibitory and activated IC receptors. The above figure is an example of BM samples from AML patients. The lower panel shows histograms for each IC receptor on each immune subset.

Panels 5, 6, 7 and 8: immune checkpoint ligands for AML patients

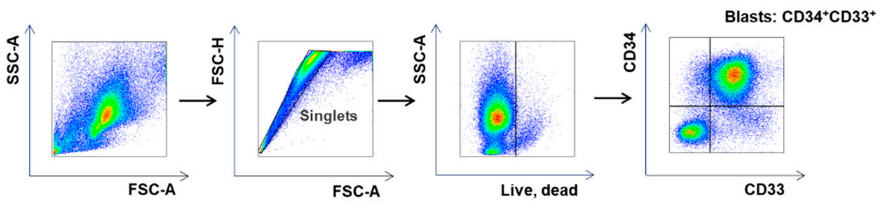

Panels 5, 6, 7, and 8 were used to detect and gate the presence of leukemic cells. The above figure is an example of BM samples from patients with AML.

Panel 9: maturation and memory phenotype for AML patients

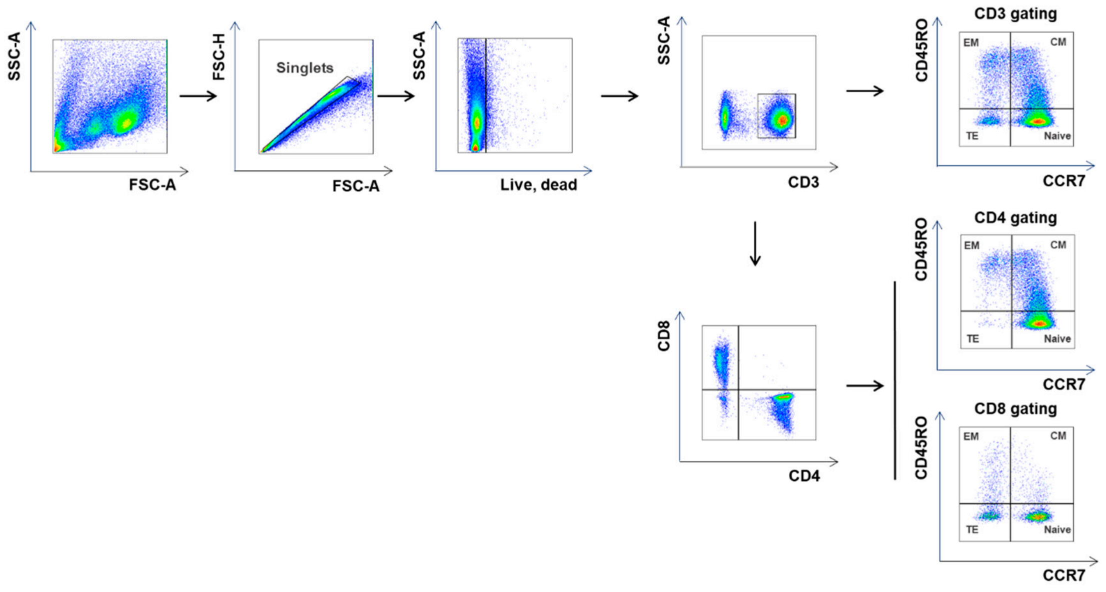

Panel 9 was used to distinguish between the maturation and memory states of T cells. The above figure is an example of BM samples from patients with AML

Panel / DNAM-1-TIGIT+CD96+/ PD-1+TIM3+ / HC BM

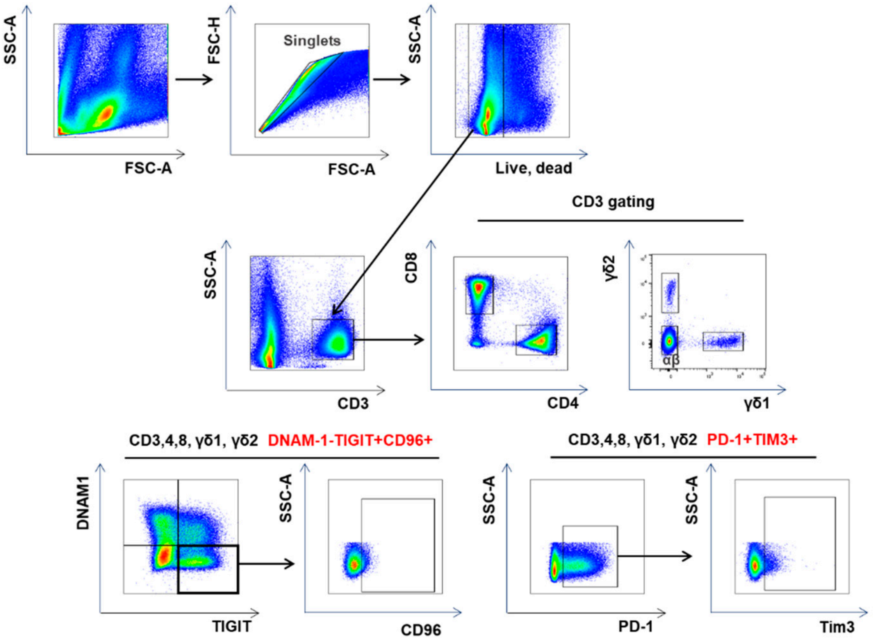

Panel / DNAM-1-TIGIT+CD96+/ PD-1+TIM3+ / AML BM

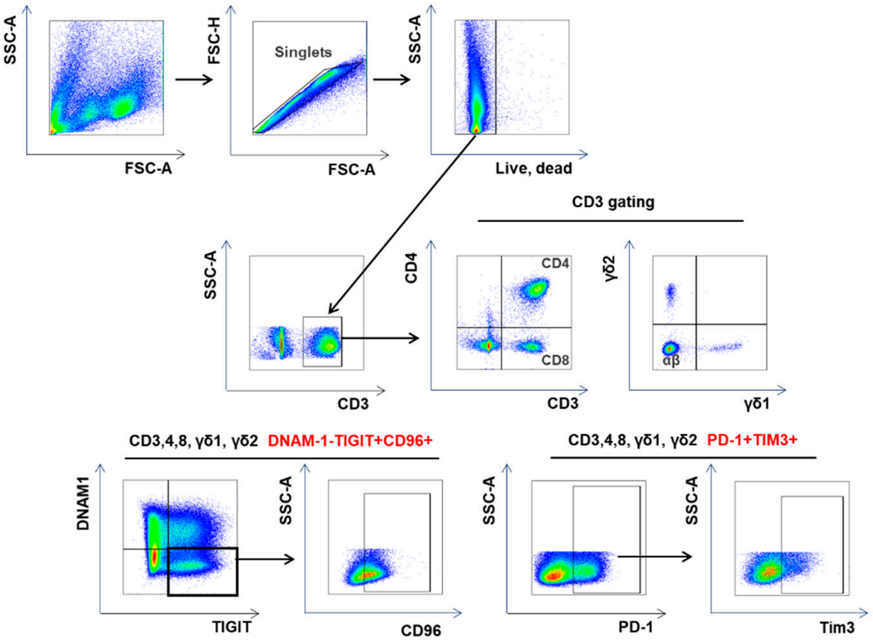

The above figures show a gating strategy of DNAM-1-TIGIT+CD96+ or PD1+TIM3+ T cell subsets from healthy controls and patients with AML.

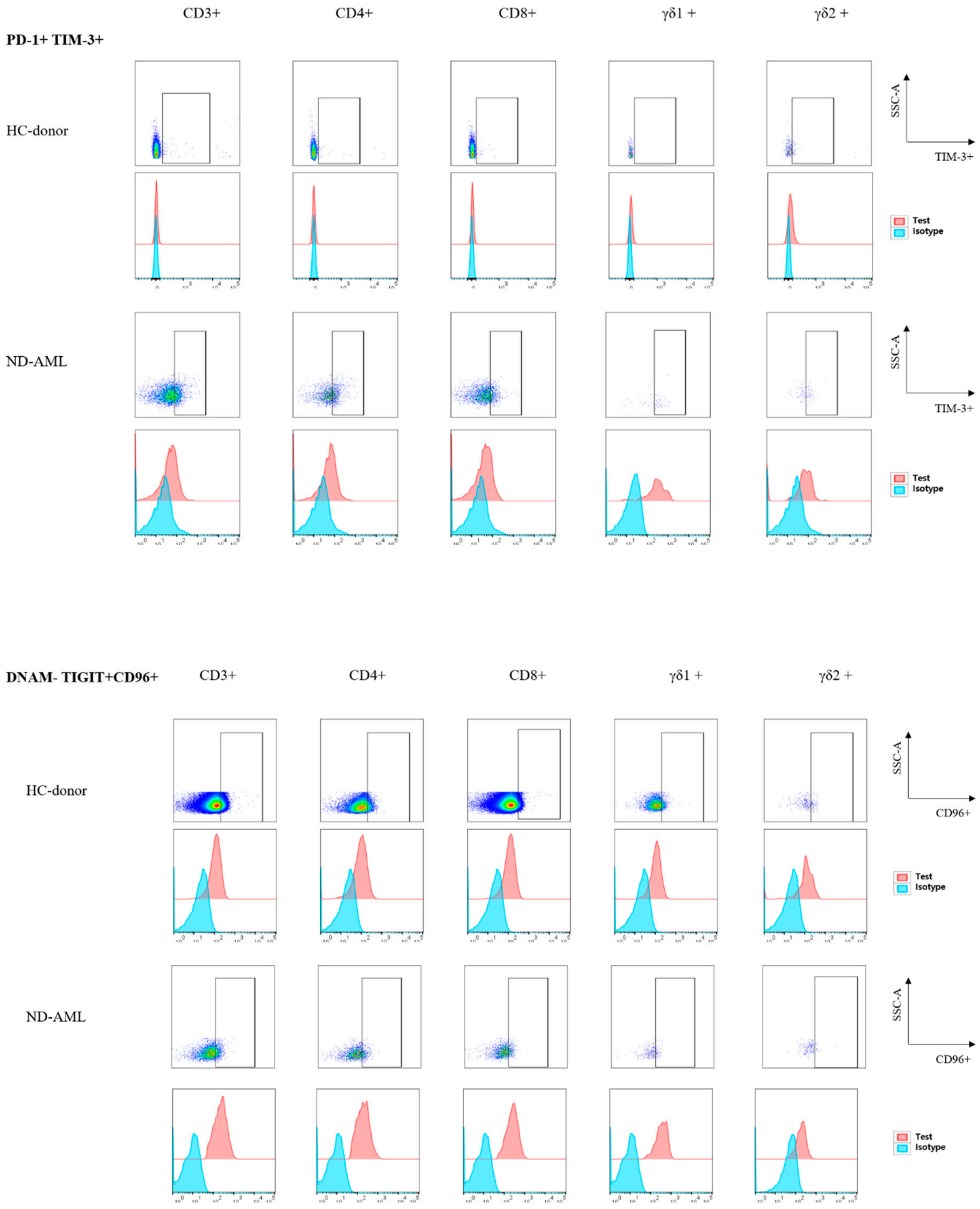

The above figures show DNAM-1-TIGIT+CD96+ or PD1+TIM3+ cells in each T cell subset from healthy controls and patients with AML.

Supplementary Methods S3. Measurable residual disease assessment with *WT1*

To analyze measurable residual disease (MRD) in patients who achieved CR, we measured *Wilms tumor gene 1* (*WT1*) transcript levels in BM samples. *WT1* levels were assessed using real-time quantitative polymerase chain reaction with a *WT1* ProfileQuant kit (Ipsogen, Marseille, France). The *WT1* gene transcripts were standardized according to *ABL1* transcript counts and expressed as the number of copies per 10^4 copies of *ABL1*. Assays were performed in triplicate to ensure the accuracy of the results. When *ABL1* quantification was unexpectedly low, the assay was repeated according to the manufacturer's instructions.
