# Supplementary material for: Characterization of the Bone Marrow Lymphoid Microenvironment and Discovery of Prognostic Immune-Related Factors in Acute Myeloid Leukemia
Source: Int J Mol Sci. 2024 Dec 4;25(23):13039. doi: 10.3390/ijms252313039 (PMC11641137; doi:10.3390/ijms252313039)
Supplement: Supplementary file 1 [file ijms-25-13039-s001.zip › Supplementary Tables - IJMS - Revised.pdf]

Supplementary Table S1. Immune checkpoint receptor expression of T cell in healthy control and newly diagnosed AML. A. The percentage of positive cells for each immune check point receptor expression within T cell subsets was compared between healthy controls and AML patients. B. Immune checkpoint receptor expression was compared by isotype-controlled mean fluorescence intensity. P values were tested by Mann-Whitney test. AML: Acute myeloid leukemia; MFI: Mean Fluorescence Intensity; ND: Newly diagnosed. †CD8+ T cells were defined as $CD4^-$ T cells among $CD3^+TCR\alpha\beta^+$ cells.

A. Proportion

Marker, % (Median, range)	$CD3^+$ T cell			$CD4^+$ T cell			$CD8^+$ T cell†		
	Healthy BM (N: 13)	ND-AML (N: 71)	*p*	Healthy BM (N: 13)	ND-AML (N: 47)	*p*	Healthy BM (N: 13)	ND-AML (N: 47)	*p*
**PD-1**	15.5 (7.0-36.3)	18.2 (3.3-47.8)	0.501	7.6 (2.4-29.5)	9.4 (2.5-56.3)	0.2868	19.6 (9.6-41.9)	21.2 (5.7-55.2)	0.7319
**TIM-3**	1.4 (0.1-12.5)	2.3 (0.2-18.7)	0.1703	0.5 (0.1-3.4)	0.6 (0.1-25.6)	0.2345	0.6 (0.3-1.2)	0.5 (0.1-11.6)	0.6258
**CTLA-4**	2.2 (0-17.1)	0.4 (0-66.2)	0.0345*	2.0 (0.1-17.9)	0.5 (0-53.7)	0.0294*	0.3 (0-2.8)	0 (0-1.4)	0.0122*
**TIGIT**	23.0 (8.7-56.2)	33.2 (0.2-72.8)	0.0368*	5.7 (1.8-22.2)	20.1 (1.4-78.9)	0.0003***	39.5 (18.4-70.7)	60 (21.7-86.8)	0.0045**
**CD112R**	15.2 (1.7-26.7)	11.2 (0.6-51.7)	0.5943	1.8 (0.5-8.4)	2.4 (0-33.6)	0.4908	22.5 (2.0-48.6)	21.3 (5.2-63.4)	0.4899
**CD96**	12.0 (0.8-19.7)	1.3 (0-60.4)	0.0032**	2.5 (0.4-9.5)	1.1 (0-68.3)	0.2562	5.8 (0.7-33.3)	0.8 (0-15.3)	<0.0001****
**DNAM-1**	49.6 (11.8-62)	56.6 (20.9-94.9)	0.0455*	56.9 (10.4-76.9)	60.1 (21.0-99.1)	0.4787	41.5 (11.9-73.8)	45.6 (18.3-85.8)	0.5245
**NKG2D**	56.9 (23.8-84.7)	43.9 (4.6-75.5)	0.0435*	9.9 (2.2-56.6)	5.2 (0.1-60.4)	0.1252	84.1 (49.7-97.4)	75.6 (41.9-94.1)	0.0396*
**NKp30**	23.3 (7.8-54.6)	4.5 (0-68.5)	0.0004***	56.5 (9.9-79.6)	21.6 (0-91)	0.0021**	4.9 (0.3-33.6)	4.6 (0.7-11.7)	0.782
**NKp44**	0.9 (0.1-10.1)	0.5 (0-55.7)	0.3741	0.4 (0-9.6)	0.3 (0-16.9)	0.497	0 (0-2.5)	0.3 (0-4.2)	0.0093**
**OX40**	1.6 (0.3-11.2)	4.1 (0.5-17.8)	0.0758	2.7 (0.5-16.6)	6.4 (0.6-32.8)	0.0174*	0.2 (0-6.1)	1.1 (0.2-17.8)	0.0481*

Marker, % (Median, range)	Vδ1 T cell			Vδ2 T cell		
	Healthy BM (N: 13)	ND-AML (N: 71)	*p*	Bone marrow (N: 13)	ND-AML (N: 71)	*p*
**PD-1**	40.6 (5.0-83.5)	48.8 (8.0-100)	0.2367	5.8 (0-17.0)	6.9 (0-47.7)	0.2679
**TIM-3**	1.7 (0-39.3)	9.8 (0.4-74.3)	0.0014**	17.0 (1.1-65.9)	12.2 (0-81.8)	0.7240
**CTLA-4**	2.4 (0-26.8)	1.4 (0-29.5)	0.8218	2.6 (0.2-26.3)	1 (0-67.1)	0.2228
**TIGIT**	69.8 (32.2-94.9)	89.8 (22.6-99.8)	0.0062**	3.1 (0-84.3)	24.0 (1.2-90.6)	0.0063*
**CD112R**	48.6 (11.9-87.8)	35.0 (2.8-83.9)	0.5767	17.6 (2.0-48.1)	14.0 (0-92.3)	0.8190
**CD96**	12.2 (1-63.5)	8.5 (0-92.7)	0.2599	24.5 (4.0-61.2)	8.1 (0-63.5)	0.0583
**DNAM-1**	21.3 (2.6-80.1)	27.0 (2.5-88.4)	0.8273	89.8 (40.6-100)	88.4 (41.6-100)	0.9455
**NKG2D**	93.7 (75.3-98.1)	87.2 (46.7-99.7)	0.2263	92.7 (72.2-98.6)	79.2 (14.7-99.7)	0.0251*
**NKp30**	13.2 (3.6-63.1)	35.2 (0-87.1)	0.0373*	44.2 (7.7-97.5)	17.4 (0-94.9)	0.0068*
**NKp44**	1.5 (0.1-6.5)	2.7 (0-70)	0.1439	1.5 (0.1-15)	2.1 (0-45.7)	0.2656

**OX40**	1.5 (0-12.6)	2.2 (0-71)	0.6628	8.5 (2.1-74.3)	15.8 (0-58.7)	0.6230	

B. MFI/Isotype

Marker, MFI/Isotype (Median, range)	CD3⁺ T cell			CD4⁺ T cell			CD8⁺ T cell†		
	Healthy BM (N: 13)	ND-AML (N: 71)	*p*	Healthy BM (N: 13)	ND-AML (N: 71)	*p*	Healthy BM (N: 13)	ND-AML (N: 71)	*p*
**PD-1**	4.0 (2.1-27.2)	6.6 (2.0-37.1)	0.0552	2.2 (1.3-21.3)	4.2 (1.0-38.5)	0.0057**	5.0 (2.2-20.3)	7.5 (2.8-53.4)	0.3707
**TIM-3**	2.7 (1.5-97.6)	2.2 (0.2-6.6)	0.0989	1.6 (0.8-3.3)	1.8 (0.1-5.5)	0.7853	2.1 (1.3-6.5)	2 (0.4-6.3)	0.1472
**CTLA-4**	1.6 (0.6-2.3)	1.5 (0.2-16.5)	0.9641	1.7 (0.5-3.4)	2.4 (0.3-13.6)	0.0426*	1.4 (0.5-2.1)	1.2 (0.2-6.0)	0.6871
**TIGIT**	16.4 (3.0-30.9)	10.6 (1.5-50.7)	0.1588	8.1 (2.0-19.8)	5.4 (1.8-16.5)	0.317	27.6 (3.3-55.4)	11.2 (2.2-56.4)	0.0784
**CD112R**	2.1 (1.3-3.7)	3.4 (1.0-14.4)	0.014*	1.1 (0.7-2.6)	2.0 (0.6-8.7)	0.0032**	3.0 (2.0-5.5)	3.7 (0.9-22.6)	0.0428*
**CD96**	2.3 (1.1-4.3)	1.6 (0.3-5.4)	0.0229*	1.9 (0.6-4.0)	1.3 (0.3-4.2)	0.0806	3.0 (1.3-5.1)	1.9 (0.4-7.6)	0.0125*
**DNAM-1**	12.8 (4.3-58.2)	16.6 (3.6-71.6)	0.0362*	12.9 (4.4-75.2)	18.1 (5.1-77.1)	0.0806	8.9 (3.6-36.3)	13.7 (3.2-73.1)	0.0605
**NKG2D**	3.4 (2.5-10.8)	2.6 (0.4-13.5)	0.0482*	0.8 (0.4-5.8)	0.7 (0.2-5.4)	0.3675	6.2 (3.1-54.2)	3.6 (0.5-28.3)	0.0102*
**NKp30**	3.1 (1.0-6)	2.1 (0.3-17.3)	0.0309*	5.5 (3.2-11.0)	4.1 (0.1-22.2)	0.1584	1.6 (0.5-4.4)	1.7 (0.5-5.5)	0.6861
**NKp44**	1.9 (0.6-5.7)	1.2 (0.4-7.1)	0.0137*	1.8 (0.6-6.6)	1.3 (0.2-8.0)	0.1263	1.7 (0.5-4.2)	1.5 (0.4-3.8)	0.2157
**OX40**	1.2 (0.1-2.2)	1.1 (0.2-4.1)	0.7186	0.9 (0.1-2.7)	0.8 (0-3.8)	0.9106	1.3 (0.1-2.0)	0.7 (0.1-2.7)	0.1252

Marker, MFI/Isotype (Median, range)	Vδ1 T cell			Vδ2 T cell		
	Healthy BM (N: 13)	ND-AML (N: 71)	*p*	Bone marrow (N: 13)	ND-AML (N: 71)	*p*
**PD-1**	8.2 (2.4-98.4)	16.2 (2.3-445.8)	0.026*	2.4 (1.7-10.3)	3.5 (0.8-14.2)	0.1156
**TIM-3**	2.6 (1.2-7.9)	3.7 (0.7-89.4)	0.0724	8.6 (2.9-602.6)	4.5 (0.8-17.3)	0.001**
**CTLA-4**	1.9 (0.6-4)	2.2 (0.2-11.4)	0.0737	1.5 (0.2-4.1)	2.1 (0.1-11.1)	0.3344
**TIGIT**	44.2 (5.3-207.6)	35.4 (4.1-126.5)	0.1167	3.9 (0.3-21.3)	5 (0.9-38.8)	0.4631
**CD112R**	5.2 (3-8.1)	7.6 (2.5-32.5)	0.0223*	2.9 (0.7-5.4)	3.5 (1.1-33.7)	0.0116*
**CD96**	3.5 (2.4-7.2)	2.7 (0.4-16)	0.1917	3.8 (2.1-8.4)	2.7 (0.5-8)	0.0124*
**DNAM-1**	4.6 (2.5-18.3)	6.2 (0.9-65.2)	0.3762	19.7 (11.8-80.3)	28.8 (3.6-104.4)	0.1796
**NKG2D**	7.8 (5.2-49)	6.8 (1.3-31.3)	0.31	6.2 (2.8-17.7)	4.5 (0.6-24.3)	0.0631
**NKp30**	2.8 (1.4-6.1)	4.9 (0.5-35.2)	0.0053**	6.6 (2.5-16.5)	3.2 (0.1-14.4)	0.0065**
**NKp44**	2.7 (1.6-25.4)	2.3 (0.5-20.2)	0.4263	1.8 (0.2-9.7)	2.5 (0.4-12.9)	0.3908
**OX40**	1.5 (0.2-2.4)	1.4 (0.1-28.4)	0.7239	2.8 (0.5-10.9)	1.9 (0.3-18.3)	0.2959

Supplementary Table S2. Comparison of Immune checkpoint expression of T cell according to disease status of AML in pooled samples. A. The percentage of positive cells for each immune check point receptor expression within 5 T cell subsets (CD3+, CD4+, CD8+, Vδ1, and Vδ2) at various disease state (ND, CR, and R/R) was compared. B. Immune checkpoint receptor expression was compared by isotype-controlled mean fluorescence intensity. P values were tested by Mann-Whitney test. AML: Acute myeloid leukemia; CR: Complete remission; MFI: Mean Fluorescence Intensity; ND: Newly diagnosed; R/R: Relapse/Refractory. †$CD8^+$ T cells were defined as $CD4^-$ T cells among $CD3^+TCR\alpha\beta^+$ cells.

A. Percentage

Marker, % (Median, range)	$CD3^+$ T cell			p		
	ND (N: 71)	CR (N: 47)	R/R (N: 40)	ND vs.CR	CR vs. R/R	ND vs. R/R
**PD-1**	2.3 (0.2-18.7)	1.4 (0.1-19.8)	3 (0.2-26.4)	0.1153	0.0167*	0.1997
**TIM-3**	0.4 (0-66.2)	0.3 (0.1-6.3)	0.6 (0-5.1)	0.4527	0.504	0.8755
**CTLA-4**	33.2 (0.2-72.8)	25.5 (3.8-71)	28.9 (4.7-80.7)	0.008**	0.1802	0.5162
**TIGIT**	11.2 (0.6-51.7)	9.9 (0.7-45.2)	7.8 (1.9-24.2)	0.9241	0.3634	0.3523
**CD112R**	1.3 (0-60.4)	0.8 (0-25.1)	3.5 (0.1-24.7)	0.3426	0.2128	0.6849
**CD96**	56.6 (20.9-94.9)	56.2 (32.2-91)	56.5 (23.1-85)	0.8727	0.3881	0.3826
**DNAM-1**	43.9 (4.6-75.5)	44.4 (10.1-73.4)	41.2 (6.1-81.9)	0.9462	0.4877	0.5121
**NKG2D**	43.9 (4.6-75.5)	44.4 (10.1-73.4)	41.2 (6.1-81.9)	0.9462	0.4877	0.5121
**NKp30**	4.5 (0-68.5)	8.6 (0.1-96.6)	18.9 (0-66.7)	0.1164	0.2025	0.0134*
**NKp44**	0.5 (0-55.7)	0.7 (0.1-12.6)	0.5 (0-6.8)	0.2703	0.2521	0.7686
**OX40**	4.1 (0.5-17.8)	3.8 (0.6-27.2)	5 (0.1-15.2)	0.786	0.993	0.756

Marker, % (Median, range)	$CD4^+$ T cell			p		
	ND (N: 71)	CR (N: 47)	R/R (N: 40)	ND vs.CR	CR vs. R/R	ND vs. R/R
**PD-1**	18.2 (3.3-47.8)	14.8 (4-92.8)	20.4 (3-53.5)	0.0192*	0.0225*	0.3878
**TIM-3**	2.3 (0.2-18.7)	1.4 (0.1-19.8)	3 (0.2-26.4)	0.1153	0.0167*	0.1997
**CTLA-4**	0.4 (0-66.2)	0.3 (0.1-6.3)	0.6 (0-5.1)	0.4527	0.504	0.8755
**TIGIT**	33.2 (0.2-72.8)	25.5 (3.8-71)	28.9 (4.7-80.7)	0.008**	0.1802	0.5162
**CD112R**	11.2 (0.6-51.7)	9.9 (0.7-45.2)	7.8 (1.9-24.2)	0.9241	0.3634	0.3523
**CD96**	1.3 (0-60.4)	0.8 (0-25.1)	3.5 (0.1-24.7)	0.3426	0.2128	0.6849
**DNAM-1**	56.6 (20.9-94.9)	56.2 (32.2-91)	56.5 (23.1-85)	0.8727	0.3881	0.3826
**NKG2D**	43.9 (4.6-75.5)	44.4 (10.1-73.4)	41.2 (6.1-81.9)	0.9462	0.4877	0.5121
**NKp30**	4.5 (0-68.5)	8.6 (0.1-96.6)	18.9 (0-66.7)	0.1164	0.2025	0.0134*
**NKp44**	0.5 (0-55.7)	0.7 (0.1-12.6)	0.5 (0-6.8)	0.2703	0.2521	0.7686
**OX40**	4.1 (0.5-17.8)	3.8 (0.6-27.2)	5 (0.1-15.2)	0.786	0.993	0.756

Marker, % (Median, range)	CD8+ T cell†			p		
	ND (N: 71)	CR (N: 47)	R/R (N: 40)	ND vs.CR	CR vs. R/R	ND vs. R/R
**PD-1**	21.2 (5.7-55.2)	16.6 (5.1-52.9)	16.3 (3.9-39.4)	0.0526	0.7014	0.1706
**TIM-3**	0.5 (0.1-11.6)	0.4 (0.1-18.2)	1.2 (0.3-3.7)	0.3635	0.0155*	0.0406*
**CTLA-4**	0 (0-1.4)	0 (0-0.4)	0 (0-0.6)	0.2063	0.5965	0.7504
**TIGIT**	60 (21.7-86.8)	50.1 (14.1-88.7)	38.5 (26.9-81.7)	0.1643	0.4088	0.0146*
**CD112R**	21.3 (5.2-63.4)	22.1 (5-44.7)	10.4 (3.3-19.5)	0.9512	0.0191*	0.0056**
**CD96**	0.8 (0-15.3)	0.2 (0-2.2)	0.9 (0.1-4.6)	0.006**	0.0564	0.517
**DNAM-1**	45.6 (18.3-85.8)	44.2 (25.9-95.8)	54.4 (18.2-86.6)	0.4678	0.0651	0.1362
**NKG2D**	75.6 (41.9-94.1)	68.1 (39.4-97.4)	69.7 (37.8-95.5)	0.0543	0.8833	0.5818
**NKp30**	4.6 (0.7-11.7)	4 (0.1-12.7)	3.7 (0.2-13.8)	0.3669	0.706	0.2842
**NKp44**	0.3 (0-4.2)	0.1 (0-9.5)	0 (0-1.5)	0.2554	0.4505	0.0775
**OX40**	1.1 (0.2-17.8)	1 (0.1-8.3)	0.1 (0-8.8)	0.7095	0.0201*	0.0224*

Marker, % (Median, range)	Vδ1 T cell			p		
	ND (N: 71)	CR (N: 47)	R/R (N: 40)	ND vs.CR	CR vs. R/R	ND vs. R/R
**PD-1**	48.8 (8-100)	41.9 (6.4-84)	48.2 (17.2-97.7)	0.3708	0.3419	0.8528
**TIM-3**	9.8 (0.4-74.3)	4.1 (0-32.1)	9.2 (0-53.5)	0.0038**	0.022*	0.8091
**CTLA-4**	1.4 (0-29.5)	0.6 (0-20.3)	0.8 (0-23.2)	0.0308*	0.3887	0.312
**TIGIT**	89.8 (22.6-99.8)	84 (5-99.5)	83 (43.5-99.2)	0.0204*	0.3186	0.2165
**CD112R**	35 (2.8-83.9)	44.8 (16.9-81.4)	30.2 (5.7-69.1)	0.0853	0.0168*	0.5548
**CD96**	8.5 (0-92.7)	8.2 (0-66.2)	11.2 (0.2-41.5)	0.7175	0.6496	0.5592
**DNAM-1**	27 (2.5-88.4)	24.6 (2.9-87.3)	24.4 (1-88.9)	0.5244	0.5644	0.8528
**NKG2D**	87.2 (46.7-99.7)	73.8 (24.4-97.3)	71.2 (12.7-99.1)	0.0006***	0.4492	0.0004***
**NKp30**	35.2 (0-87.1)	39.2 (1.7-89.9)	19.8 (0-84.7)	0.4055	0.052	0.1528
**NKp44**	2.7 (0-70)	0.9 (0.1-15.3)	1.2 (0-52)	0.0117*	0.5849	0.0271*
**OX40**	2.2 (0-71)	4 (0-46.3)	3.2 (0-63.9)	0.0634	0.343	0.5539

Marker, % (Median, range)	Vδ2 T cell			p		
	ND (N: 71)	CR (N: 47)	R/R (N: 40)	ND vs.CR	CR vs. R/R	ND vs. R/R
**PD-1**	6.9 (0-47.7)	7.3 (0.9-55.1)	9.3 (1.2-56.1)	0.9599	0.7458	0.6325
**TIM-3**	12.2 (0-81.8)	7.6 (0.5-74.5)	13.1 (1-91.9)	0.1191	0.0193*	0.3685
**CTLA-4**	1 (0-67.1)	0.5 (0-47.8)	0.5 (0-20.9)	0.1982	0.4371	0.7105
**TIGIT**	24 (1.2-90.6)	10.6 (0.1-76.4)	14.4 (0.5-82.8)	0.0017**	0.1908	0.1634
**CD112R**	14 (0-92.3)	14.9 (2.8-60.1)	7.8 (0.7-29.3)	0.3473	0.0004***	0.0213*
**CD96**	8.1 (0-63.5)	4.2 (0-53.3)	11.5 (0.2-56.2)	0.0868	0.1217	0.8301

**DNAM-1**	88.4 (41.6-100)	86.8 (12.1-99.1)	87.7 (50.7-99.1)	0.4959	0.8444	0.4613
**NKG2D**	79.2 (14.7-99.7)	63 (9.4-97.8)	54.4 (16.8-97.8)	0.0015**	0.3151	0.0013**
**NKp30**	17.4 (0-94.9)	9.8 (0-80.5)	12.1 (0-74.2)	0.1605	0.3165	0.8277
**NKp44**	2.1 (0-45.7)	1 (0-28.4)	0.6 (0-20.7)	0.0062**	0.2972	0.0049**
**OX40**	15.8 (0-58.7)	21.6 (0.5-69.7)	6.5 (0-64.1)	0.0311*	0.0009***	0.0725

B. MFI/Isotype

**Marker, MFI/Isotype (Median, range)**	**CD3⁺ T cell**			**p**		
	**ND (N: 71)**	**CR (N: 47)**	**R/R (N: 40)**	**ND vs.CR**	**CR vs. R/R**	**ND vs. R/R**
**PD-1**	6.6 (2-37.1)	3.7 (1-16)	6.1 (1.7-36.9)	<0.0001****	0.0002***	0.3153
**TIM-3**	2.2 (0.2-6.6)	2.2 (0.9-6.4)	2.6 (0.9-6.2)	0.3809	0.2549	0.0449*
**CTLA-4**	1.5 (0.2-16.5)	1.9 (0.6-5.1)	1.5 (0.6-3.5)	0.1324	0.0609	0.9974
**TIGIT**	10.6 (1.5-50.7)	8.6 (1.8-28.4)	11.9 (1.4-69.7)	0.1231	0.0502	0.4093
**CD112R**	3.4 (1-14.4)	2.8 (1-12.2)	2.8 (1.1-7.3)	0.1306	0.3776	0.5339
**CD96**	1.6 (0.3-5.4)	1.6 (0.3-33.4)	2.3 (1.1-5.9)	0.7028	0.0139*	0.0104*
**DNAM-1**	16.6 (3.6-71.6)	19.3 (6.4-157)	25.6 (4.7-64.1)	0.2896	0.0632	0.0199*
**NKG2D**	2.6 (0.4-13.5)	3.5 (0.4-10.7)	2.5 (0.4-16.8)	0.1751	0.257	0.7905
**NKp30**	2.1 (0.3-17.3)	2.7 (0.5-8.6)	2.9 (0.5-8.3)	0.0997	0.7078	0.0627
**NKp44**	1.2 (0.4-7.1)	1.6 (0.5-6)	1.5 (0.6-3.9)	0.0061**	0.7422	0.0068**
**OX40**	1.1 (0.2-4.1)	1.1 (0.1-5.7)	1.4 (0.1-12.8)	0.8632	0.2571	0.191

**Marker, MFI/Isotype (Median, range)**	**CD4⁺ T cell**			**p**		
	**ND (N: 71)**	**CR (N: 47)**	**R/R (N: 40)**	**ND vs.CR**	**CR vs. R/R**	**ND vs. R/R**
**PD-1**	4.2 (1-38.5)	2.7 (0.9-12.1)	4.4 (1.2-18.4)	0.0002***	0.031*	0.6102
**TIM-3**	1.8 (0.1-5.5)	1.8 (0.5-5.1)	2.5 (0.4-6.8)	0.2616	0.1986	0.033*
**CTLA-4**	2.4 (0.3-13.6)	2.6 (0.7-6.9)	2.6 (0.7-5.1)	0.5426	0.7416	0.424
**TIGIT**	5.4 (1.8-16.5)	4.8 (1.1-46.1)	6 (0.9-99.1)	0.1826	0.0925	0.5528
**CD112R**	2 (0.6-8.7)	1.8 (0.3-9.5)	2.1 (0.4-6.8)	0.1941	0.2103	0.9545
**CD96**	1.3 (0.3-4.2)	1.3 (0.2-30.8)	2.3 (0.5-6.5)	0.6502	0.0431*	0.008**
**DNAM-1**	18.1 (5.1-77.1)	21.4 (7.3-120.9)	26.1 (5.7-56.4)	0.3202	0.2309	0.1803
**NKG2D**	0.7 (0.2-5.4)	0.9 (0.2-5.1)	0.8 (0.2-3.6)	0.1068	0.5317	0.4415
**NKp30**	4.1 (0.1-22.2)	3.1 (0.4-10.8)	5.1 (0.9-21.1)	0.1799	0.0156*	0.451
**NKp44**	1.3 (0.2-8)	1.6 (0.3-12.2)	2 (0.6-6.4)	0.0519	0.2439	0.0034**
**OX40**	0.8 (0-3.8)	1.2 (0.1-6.8)	1 (0.2-4)	0.1026	0.5922	0.4241

Marker, MFI/Isotype (Median, range)	CD8^{+} T cell[†]			p		
	ND (N: 71)	CR (N: 47)	R/R (N: 40)	ND vs.CR	CR vs. R/R	ND vs. R/R
**PD-1**	7.5 (2.8-53.4)	4.4 (0.8-22)	6 (2.4-16.5)	0.0002***	0.0057**	0.3206
**TIM-3**	2 (0.4-6.3)	2.4 (0.7-6.8)	2.8 (1-5.7)	0.0834	0.1503	0.0079**
**CTLA-4**	1.2 (0.2-6)	1.2 (0.4-2.8)	1.2 (0.4-2.3)	0.5868	0.2858	0.208
**TIGIT**	11.2 (2.2-56.4)	12.3 (2.4-35.1)	12.7 (1.4-81.5)	0.3047	0.2512	0.801
**CD112R**	3.7 (0.9-22.6)	3.4 (1.2-15.7)	3.1 (1.2-5.7)	0.1364	0.2395	0.0058**
**CD96**	1.9 (0.4-7.6)	1.6 (0.3-38.1)	2.4 (1-5.4)	0.8252	0.0517	0.0836
**DNAM-1**	13.7 (3.2-73.1)	17.7 (4.5-189.9)	24.9 (4.3-46.1)	0.1058	0.1698	0.0311*
**NKG2D**	3.6 (0.5-28.3)	6.3 (1.1-20.5)	4.2 (2.1-23.9)	0.0552	0.2442	0.3327
**NKp30**	1.7 (0.5-5.5)	1.8 (0.6-5.9)	1.5 (0.6-8.1)	0.6212	0.2949	0.1136
**NKp44**	1.5 (0.4-3.8)	1.7 (0.5-4.1)	1.7 (0.5-4.9)	0.1526	0.9957	0.1791
**OX40**	0.7 (0.1-2.7)	1 (0.2-4.2)	0.8 (0.1-3.8)	0.0454*	0.3695	0.5558

Marker, MFI/Isotype (Median, range)	Vδ1 T cell			p		
	ND (N: 71)	CR (N: 47)	R/R (N: 40)	ND vs.CR	CR vs. R/R	ND vs. R/R
**PD-1**	16.2 (2.3-445.8)	8.3 (1.9-32.6)	12.3 (5-60.3)	0.0105*	0.0405*	0.4271
**TIM-3**	3.7 (0.7-89.4)	3.5 (1.5-10.7)	4.1 (1.8-14.8)	0.4419	0.1276	0.4805
**CTLA-4**	2.2 (0.2-11.4)	2 (0.5-7.2)	2.4 (0-8)	0.5281	0.7247	0.8353
**TIGIT**	35.4 (4.1-126.5)	34.5 (6-85.3)	36.5 (2.8-248.1)	0.2729	0.2358	0.6939
**CD112R**	7.6 (2.5-32.5)	5.6 (2.2-35.3)	6.3 (3.6-20)	0.1322	0.5022	0.4532
**CD96**	2.7 (0.4-16)	2.5 (0.4-49.7)	3.1 (1.5-7.3)	0.9155	0.1886	0.3439
**DNAM-1**	6.2 (0.9-65.2)	6.9 (1.4-145.7)	9.5 (0.6-64.1)	0.7814	0.192	0.1593
**NKG2D**	6.8 (1.3-31.3)	8.4 (1.2-45.1)	5.1 (1.3-18.7)	0.301	0.0616	0.3221
**NKp30**	4.9 (0.5-35.2)	7 (1.2-58.5)	4.6 (1.3-30)	0.2017	0.1467	0.5992
**NKp44**	2.3 (0.5-20.2)	2.4 (0.5-21.1)	2.4 (0.9-11.2)	0.6121	0.8851	0.6692
**OX40**	1.4 (0.1-28.4)	1.6 (0.1-9.3)	1.6 (0-8.1)	0.6809	0.8761	0.9246

Marker, MFI/Isotype (Median, range)	Vδ2 T cell			p		
	ND (N: 71)	CR (N: 47)	R/R (N: 40)	ND vs.CR	CR vs. R/R	ND vs. R/R
**PD-1**	3.5 (0.8-14.2)	2.6 (0.5-9.7)	3.4 (1.1-20.7)	0.0699	0.0475*	0.6816
**TIM-3**	4.5 (0.8-17.3)	4.5 (1.6-15.7)	5.4 (1.9-43.7)	0.4651	0.0925	0.018*
**CTLA-4**	2.1 (0.1-11.1)	2.1 (0.6-23.7)	2.5 (0.7-7.8)	0.7946	0.6388	0.5779
**TIGIT**	5 (0.9-38.8)	4 (0.5-25.3)	5.3 (0.7-77.6)	0.1191	0.2381	0.9637
**CD112R**	3.5 (1.1-33.7)	3.1 (1.2-21.6)	2.9 (0.5-7.4)	0.0957	0.8659	0.0624
**CD96**	2.7 (0.5-8)	1.9 (0.4-35.5)	3.2 (1.2-8.1)	0.3621	0.0347*	0.2399

**DNAM-1**	28.8 (3.6-104.4)	30.6 (7.5-190.1)	41.8 (5.5-101.3)	0.4945	0.308	0.1892
**NKG2D**	4.5 (0.6-24.3)	6.3 (0.9-16.1)	3.7 (0.8-27.2)	0.1915	0.0799	0.7042
**NKp30**	3.2 (0.1-14.4)	3 (0.4-33.5)	4 (1-11.7)	0.8168	0.0887	0.4059
**NKp44**	2.5 (0.4-12.9)	2.2 (0.3-9.5)	2.2 (0.9-3.7)	0.6994	0.7769	0.5778
**OX40**	1.9 (0.3-18.3)	2.7 (0.1-13.4)	2 (0.4-7.3)	0.0379*	0.0281*	0.5077

Supplementary Table S3. Comparison of Immune checkpoint expression of T cell according to disease status of AML in paired samples. A. The percentage of positive cells for each immune check point receptor expression within 5 T cell subsets (CD3+, CD4+, CD8+, Vδ1, and Vδ2) at various disease state (ND, CR, and R/R) was compared. B. Immune checkpoint receptor expression was compared by isotype-controlled mean fluorescence intensity. P values were tested by Wilcoxon signed-rank test. When comparing CR vs. Rel and ND vs. R/R, only 20 and 26 paired samples used for testing, respectively. AML: Acute myeloid leukemia; CR: Complete remission; MFI: Mean Fluorescence Intensity; ND: Newly diagnosed; Rel: Relapse; R/R: Relapse/Refractory. †CD8^{+} T cells were defined as CD4^{-} T cells among CD3^{+}TCRαβ^{+} cells.

A. Percentage

Marker, % (Median, range)	CD3⁺ T cell								
	ND (N: 38)	CR (N: 38)	p	CR (N: 20)	Rel (N: 20)	p	ND (N: 26)	R/R (N: 26)	p
**PD-1**	15.9 (3.3-47.8)	14.8 (4-92.8)	0.1126	11.9 (4-92.8)	17.1 (3.9-49.9)	0.0296*	16.9 (8.5-31)	19.6 (3-49.9)	0.8823
**TIM-3**	1.3 (0.2-14.3)	1 (0.1-19.8)	1	2.7 (0.1-19.8)	3.2 (0.2-13.5)	0.3045	3.6 (0.4-14.3)	2.9 (0.2-13.5)	0.2411
**CTLA-4**	0.5 (0-6)	0.3 (0.1-6.3)	0.2578	0.3 (0.1-5.9)	0.5 (0-3.1)	0.4666	0.5 (0-6)	0.5 (0-2.6)	0.0551
**TIGIT**	33.4 (5.8-67.2)	25.2 (5.4-71)	0.0544	22.9 (3.8-42.2)	27.7 (10.4-72.4)	0.0056**	32.9 (20.9-60)	30.3 (10.9-66.8)	0.3666
**CD112R**	11.7 (0.6-51.7)	12 (0.7-43.5)	0.5691	5.6 (0.7-43.5)	6 (2.3-21.8)	0.2769	6.9 (1.8-20.8)	7.8 (2.3-24.2)	0.1046
**CD96**	0.7 (0-15.6)	0.6 (0-25.1)	0.1161	4 (0-25.1)	1.3 (0.1-24.7)	1	4.3 (0.2-16.2)	0.9 (0.1-12.6)	0.0052**
**DNAM-1**	56.6 (23-79.3)	54.5 (32.2-91)	0.1815	61.5 (33.3-91)	56.3 (23.1-79.9)	0.2043	51.7 (20.9-77.8)	55.8 (23.1-79.9)	0.3967
**NKG2D**	47.1 (10.9-75.5)	46.2 (10.1-73.4)	0.5412	34.7 (10.1-70.9)	31.3 (6.1-77.2)	0.9854	43.2 (10.9-69.9)	41.4 (6.1-81.1)	0.731
**NKp30**	5.5 (0-52.3)	7.3 (0.1-96.6)	0.8341	16.9 (0.1-96.6)	8.8 (0-60.7)	0.0441*	7.2 (0-64.4)	6.9 (0-66.7)	0.438
**NKp44**	0.7 (0.1-14)	0.6 (0.1-12.6)	0.6657	0.6 (0.1-5.9)	0.6 (0-6.6)	0.9839	0.2 (0.1-14)	0.5 (0-6.6)	1
**OX40**	3.4 (0.5-17.8)	3.8 (0.6-27.2)	0.4317	5.3 (1.2-20.3)	2.8 (0.1-15.2)	0.0759	4.4 (1.6-10.2)	5.1 (0.1-15.2)	0.8334

Marker, % (Median, range)	CD4⁺ T cell								
	ND (N: 38)	CR (N: 38)	p	CR (N: 20)	Rel (N: 20)	p	ND (N: 26)	R/R (N: 26)	p
**PD-1**	9.3 (2.5-56.3)	7.6 (1.5-19.6)	0.0857	7.3 (1.5-14.5)	12.2 (3.9-51.9)	0.0353*	10 (4.3-38.6)	11.3 (1.9-51.9)	0.2979
**TIM-3**	0.5 (0.1-10.2)	0.3 (0-12.1)	0.6244	0.4 (0-12.1)	1.3 (0.1-10.9)	0.3942	0.8 (0.2-14.2)	1 (0.1-10.9)	0.5966
**CTLA-4**	0.8 (0-13.1)	0.3 (0-10.8)	0.1042	0.3 (0-3.1)	0.4 (0.1-4.3)	0.6387	0.9 (0.1-13.1)	0.3 (0.1-5)	0.3225
**TIGIT**	20.1 (1.4-69.8)	14.3 (3.8-99.3)	0.0815	12.7 (2.6-22.7)	16.7 (7.8-51.2)	0.0103*	20.1 (11.9-30.4)	14.5 (6.4-51.2)	0.1928
**CD112R**	4.1 (0.1-33.6)	4.3 (0.2-35.2)	0.8966	1.5 (0.2-8.3)	1.2 (0.3-12.5)	0.9341	0.8 (0.1-7.2)	2.6 (0.3-12.5)	0.0443*
**CD96**	0.4 (0-24.2)	0.4 (0-22.6)	0.3997	4.5 (0-22.6)	1.4 (0-26.9)	0.6387	5.4 (0.2-24.2)	5.2 (0-26.9)	0.2744
**DNAM-1**	65.2 (22.1-88)	56.2 (25.4-89.2)	0.2639	61.7 (25.4-80)	62 (23.8-88.8)	0.5995	60.9 (26.7-88)	59.2 (32.3-88.8)	0.8603
**NKG2D**	8.6 (0.2-60.4)	16.9 (0.5-59)	0.4698	2.9 (0.2-34.8)	3.9 (0.1-78.5)	0.8904	2.1 (0.2-16.9)	3.7 (1.6-78.5)	0.011*
**NKp30**	11.8 (0-61.8)	6.7 (0-66.3)	0.1645	7.1 (0-86.2)	16 (0.3-63.7)	0.5614	35.8 (1.2-75.8)	40.4 (0.3-68.7)	0.9399
**NKp44**	0.6 (0-15.2)	0.3 (0-23.7)	0.2376	0.2 (0-1.1)	0.2 (0-6.4)	0.978	0.2 (0-15.2)	0.3 (0.1-6.4)	0.0649
**OX40**	4.8 (0.6-32.8)	6.2 (1.8-54.2)	0.3388	4.8 (1.4-23.7)	5.7 (0.5-20.5)	0.4212	6.4 (2.5-24.5)	7.5 (0.5-20.5)	0.6322

Marker, % (Median, range)	CD8^{+} T cell†								
	ND (N: 38)	CR (N: 38)	p	CR (N: 20)	Rel (N: 20)	p	ND (N: 26)	R/R (N: 26)	p
**PD-1**	18.2 (10.7-55.2)	18.6 (8.6-52.9)	0.3465	12.5 (5.1-26.5)	8.2 (3.9-28.3)	0.8125	15.0 (10.7-25.2)	19.1 (3.9-28.3)	1
**TIM-3**	0.5 (0.2-8.8)	0.4 (0.1-18.2)	0.8617	0.7 (0.1-1.4)	0.7 (0.3-2.2)	0.6250	0.6 (0.3-2.5)	2.2 (0.3-2.7)	0.4375
**CTLA-4**	0 (0-1.4)	0 (0-0.4)	0.0898	0 (0-0.1)	0 (0-0.6)	1.0	0 (0-0.4)	0 (0-0.6)	0.4375
**TIGIT**	53.4 (36.6-86.8)	52.6 (14.1-88.7)	0.5180	38 (29.3-55.8)	30 (26.9-81.7)	0.8125	60.0 (36.6-75.1)	38.9 (30-81.7)	0.3125
**CD112R**	20.4 (5.2-63.4)	22.6 (5-44.7)	0.3529	9.8 (7.1-37.4)	10.4 (7.2-16.7)	0.8125	13.0 (7.3-22.9)	8.0 (3.3-14.3)	0.1250
**CD96**	0.8 (0-15.3)	0.3 (0.1-2.2)	0.0840	0.3 (0-1.1)	0.9 (0.2-4.6)	0.3125	0.9 (0.1-15.3)	0.3 (0.1-1.0)	0.3125
**DNAM-1**	40.1 (18.3-75.9)	44.9 (25.9-57.7)	0.8900	44.9 (33.2-51)	45.5 (18.2-86.6)	0.4375	55.2 (39-75.9)	68.2 (45.5-86.6)	0.4375
**NKG2D**	73.8 (41.9-87.2)	68.1 (39.4-97.4)	0.4874	69 (63.7-97.4)	50 (37.8-69.7)	0.1362	72.1 (42.5-92.5)	69.0 (37.8-95.5)	0.6250
**NKp30**	4.9 (0.7-11.7)	4.7 (0.1-12.7)	0.4307	5.5 (1.4-11.3)	4.3 (1.9-13.8)	0.8125	4.9 (1.1-6.5)	1.9 (0.2-4.3)	0.0625
**NKp44**	0.3 (0-4.2)	0.3 (0-9.5)	0.6441	0 (0-0.3)	0.1 (0-1.5)	0.1875	0.1 (0-0.2)	0 (0-0.6)	1.0
**OX40**	1.1 (0.2-17.8)	0.9 (0.2-8.3)	0.7049	0.6 (0.1-5.8)	0.1 (0-8.8)	0.6250	0.2 (0.2-1.6)	0.1 (0-0.5)	0.6250

Marker, % (Median, range)	Vδ1 T cell								
	ND (N: 38)	CR (N: 38)	p	CR (N: 20)	Rel (N: 20)	p	ND (N: 26)	R/R (N: 26)	p
**PD-1**	42.1 (8-95.1)	40.7 (6.4-84)	0.5608	40.5 (6.4-83.5)	45.3 (17.2-88)	0.3519	50.9 (8-89.4)	41 (17.2-88)	0.2076
**TIM-3**	4.8 (0.4-42.6)	3.3 (0-32.1)	0.4000	4.9 (0-32.1)	7.3 (0.3-16.1)	0.1046	13.2 (1-31.5)	10.3 (0-30.3)	0.0951
**CTLA-4**	2 (0-25.7)	0.5 (0-20.3)	0.0012**	0.5 (0-11)	0.4 (0-7.9)	0.8999	1.3 (0-29.5)	1 (0-10.3)	0.3633
**TIGIT**	88.2 (45.4-99.8)	84.5 (25.4-96.9)	0.1519	80.4 (5-96.1)	77.2 (43.5-98.2)	0.8999	91.6 (56.9-99.5)	87.7 (54.5-98.2)	0.0425*
**CD112R**	37.6 (6.3-77.1)	44.4 (17.2-81.4)	0.0329*	35.9 (16.9-73.4)	31.3 (12.5-68.6)	0.1514	31.1 (6.7-64.7)	33.2 (11.8-68.6)	0.7609
**CD96**	1.9 (0-52.5)	6.1 (0-66.2)	0.8987	21.6 (0-66.2)	5.3 (0.2-26.7)	0.0830	17.5 (0.8-55.9)	7.8 (0.2-39)	0.0107*
**DNAM-1**	29.8 (3.3-63.3)	23.5 (2.9-87.3)	0.0636	26.5 (2.9-87.3)	25.4 (1-74.4)	0.3484	28.8 (3.3-75.8)	23.2 (1-71.9)	0.8996
**NKG2D**	82 (48.8-99.7)	73.2 (30.5-97.3)	0.0039**	80.7 (30.5-97.3)	62.6 (12.7-97.1)	0.1591	83.3 (46.7-98.8)	75.2 (12.7-98.5)	0.0126*
**NKp30**	44 (0-87.1)	44.3 (1.7-89.9)	0.8754	27.2 (4.4-88.2)	28.9 (0-84.7)	0.7049	29.7 (0-82.5)	28.9 (0-84.7)	0.1322
**NKp44**	3.9 (0-70)	1.2 (0.1-15.3)	0.0503	0.9 (0.1-13.2)	0.6 (0-52)	0.8603	3.9 (0-34.1)	1.2 (0-52)	0.1434
**OX40**	3.1 (0-62.7)	3.6 (0-46.3)	0.2259	5 (0.4-46.3)	3.8 (0-63.9)	0.3484	2.3 (0-36.2)	3.8 (0-63.9)	0.0269*

Marker, % (Median, range)	Vδ2 T cell								
	ND (N: 38)	CR (N: 38)	p	CR (N: 20)	Rel (N: 20)	p	ND (N: 26)	R/R (N: 26)	p
**PD-1**	4.9 (0-47.7)	8.3 (0.9-55.1)	0.3516	5.2 (0.9-19.8)	7.1 (1.2-27.5)	0.2522	7 (0-39.8)	8.2 (1.2-27.5)	0.6010
**TIM-3**	6 (0-81.8)	4.7 (0.5-74.5)	1.0	13.4 (0.7-74.5)	19.6 (1-91.9)	0.9399	25.9 (0.8-74.9)	18 (1-91.9)	0.1695
**CTLA-4**	0.9 (0-67.1)	0.6 (0-47.8)	0.4519	0.4 (0-47.8)	0.5 (0-7.8)	0.8999	1.1 (0-67.1)	1.6 (0-20.9)	0.4954
**TIGIT**	16.9 (1.7-89.1)	13.3 (0.6-76.4)	0.0182*	7.1 (0.1-36.5)	8.3 (0.5-55.6)	0.1743	19.2 (1.7-67.2)	14.4 (0.5-80.4)	0.7712

**CD112R**	19 (0-70.3)	17 (2.8-60.1)	0.8613	8.9 (2.8-33.6)	7.6 (0.7-22)	0.5879	6.2 (0-25.1)	8.7 (0.7-25)	0.2524
**CD96**	3.2 (0-38.4)	1.3 (0-53.3)	0.5317	18.9 (0.1-53.3)	3.7 (0.2-33.3)	0.0942	25 (1.5-58.7)	7 (0.2-56.2)	0.0151*
**DNAM-1**	90.1 (47.8-99.5)	85.8 (12.1-99.1)	0.027*	91.7 (12.1-99.1)	91.5 (54.2-99.1)	0.3133	89.7 (53.2-98.8)	86.6 (50.7-99.1)	0.2752
**NKG2D**	76.2 (14.7-99.7)	62.1 (29-97.8)	0.021*	70.6 (29-97.8)	46.6 (16.8-95.7)	0.0505	75.8 (14.7-99.7)	61 (20.7-97.8)	0.0081**
**NKp30**	11.1 (0-79.3)	10.8 (0-80.5)	0.0737	13.4 (0-80.5)	11 (0-33.9)	0.8999	25 (0-64.6)	15.2 (0-74.2)	0.4751
**NKp44**	2 (0-45.7)	1.1 (0-28.4)	0.0094**	0.2 (0-5.5)	0.2 (0-10.5)	0.5791	1.9 (0-33.1)	0.8 (0-13.3)	0.1840
**OX40**	16 (0-58.7)	21.5 (0.5-69.7)	0.0678	17.2 (0.5-69.7)	5.8 (0-64.1)	0.0934	14.7 (0-56.8)	8.4 (0-64.1)	0.8456

B. MFI/Isotype

**Marker, MFI/Isotype (Median, range)**	**CD3$^+$ T cell**								
	**ND (N: 38)**	**CR (N: 38)**	**p**	**CR (N: 20)**	**Rel (N: 20)**	**p**	**ND (N: 26)**	**R/R (N: 26)**	**p**
**PD-1**	5.3 (2-18.4)	3.6 (1.8-16)	<0.0001****	3.6 (1.8-16)	6.1 (2.1-17.9)	0.0602	5.9 (3.7-15.5)	5.6 (1.7-36.9)	0.1199
**TIM-3**	2 (0.2-5)	2.2 (0.9-6.4)	0.0644	2.7 (0.9-6.4)	2.7 (1.4-6.2)	0.8906	2.5 (0.2-5.3)	2.3 (1.4-5.1)	0.5077
**CTLA-4**	1.6 (0.2-4.7)	1.9 (0.6-5.1)	0.4891	2.1 (0.6-4.2)	1.6 (0.6-3.5)	0.0602	1.3 (0.2-4.7)	1.5 (0.6-3.5)	0.684
**TIGIT**	6.7 (1.5-40.1)	8.2 (2.1-28.4)	0.7158	8.9 (2.3-17.1)	12.6 (2.1-69.7)	0.0108*	13.4 (3.6-30)	10.5 (2.1-69.7)	0.1730
**CD112R**	3.2 (1-12.4)	2.8 (1-6.9)	0.0698	2.7 (1-12.2)	2.7 (1.1-7.3)	0.2769	2.8 (1-7.2)	2.8 (1.9-6.5)	1.0
**CD96**	1.3 (0.3-5.1)	1.3 (0.4-33.4)	1	2.3 (0.6-33.4)	2.3 (1.1-5.9)	0.5995	1.9 (0.8-5.4)	1.9 (1.1-3.5)	0.3484
**DNAM-1**	15.3 (4.1-56.1)	18.4 (7.8-157)	0.414	21.3 (8.3-157)	19.9 (4.7-57.3)	0.3736	16.6 (4.1-60.7)	25.5 (4.7-57.3)	0.3810
**NKG2D**	1.9 (0.6-13.5)	2.8 (0.4-10.7)	0.1893	3.6 (1.1-9.8)	2.1 (0.4-11.4)	0.0042**	2.4 (0.6-8.9)	2.3 (0.4-11.4)	0.5879
**NKp30**	2.2 (0.3-17.3)	2.6 (0.5-8.6)	0.3507	3.3 (0.6-8.6)	2.6 (0.5-8.3)	0.2753	2.1 (0.3-7.9)	2.8 (0.5-6.8)	0.8949
**NKp44**	1.2 (0.5-3.6)	1.4 (0.6-6)	0.0404*	2 (0.8-6)	1.6 (0.6-3.9)	0.2253	1.2 (0.5-3.6)	1.4 (0.6-3.9)	0.7915
**OX40**	0.9 (0.2-4.1)	1.1 (0.1-4.2)	0.6916	1.2 (0.1-5.7)	1.4 (0.1-12.8)	0.3529	0.9 (0.2-2.9)	1.2 (0.1-3.9)	0.4948

**Marker, MFI/Isotype (Median, range)**	**CD4$^+$ T cell**								
	**ND (N: 38)**	**CR (N: 38)**	**p**	**CR (N: 20)**	**Rel (N: 20)**	**p**	**ND (N: 26)**	**R/R (N: 26)**	**p**
**PD-1**	3.7 (1-38.5)	2.5 (1.3-8.4)	0.0041**	2.5 (1.3-4.1)	4.4 (1.2-18.4)	0.0302*	4.4 (1.9-10.8)	2.8 (1.3-12.3)	0.1439
**TIM-3**	1.7 (0.1-3.9)	1.8 (0.5-5.1)	0.2642	1.5 (0.5-5.1)	2.4 (0.8-6.2)	0.2769	1.5 (0.1-5.5)	2.1 (0.8-5.5)	0.4637
**CTLA-4**	2.6 (0.8-6.4)	2.7 (0.7-6.9)	0.7966	2.5 (0.7-6.3)	2.6 (1.4-5.1)	0.7197	1.6 (0.6-6.4)	2.7 (0.7-5.1)	0.1354
**TIGIT**	4.5 (1.8-16.5)	3.5 (1.4-46.1)	0.2799	4.7 (1.1-9.3)	4.9 (0.9-29.9)	0.2078	6.8 (2-14.5)	4.1 (0.9-99.1)	0.1439
**CD112R**	2 (0.6-7.5)	1.8 (0.3-5)	0.0214*	1.7 (0.3-9.5)	1.6 (0.4-3.9)	0.8904	1.7 (0.6-7.1)	2.2 (0.4-6.8)	0.6685
**CD96**	1.2 (0.3-4.2)	1.1 (0.3-4.1)	0.9529	1.7 (0.4-7.8)	2.3 (0.5-6.5)	0.9341	1.4 (0.7-3.9)	1.9 (0.5-3.5)	0.8603
**DNAM-1**	16.3 (5.1-77.1)	20.4 (7.6-87.4)	0.3483	20.3 (9-56.5)	22 (5.7-56.4)	0.8904	17.8 (5.2-55.2)	23 (5.9-56.4)	0.8209
**NKG2D**	0.8 (0.2-5.4)	0.8 (0.2-5.1)	0.1937	1.1 (0.4-5.1)	0.7 (0.2-3.6)	0.0210*	0.7 (0.2-2.3)	0.8 (0.2-3.6)	0.2734
**NKp30**	2.3 (0.1-22.2)	2.4 (0.5-5.7)	0.2687	3.1 (0.5-9.4)	3.2 (0.9-21.1)	0.4543	5.2 (0.3-11.5)	5.3 (0.9-8.5)	0.2312
**NKp44**	1.1 (0.2-3.5)	1.3 (0.3-12.2)	0.0859	1.7 (0.3-3.4)	1.9 (0.6-6.4)	0.8469	1.3 (0.5-3.2)	2 (0.6-6.4)	0.0507
**OX40**	0.9 (0-3.8)	1 (0.1-6.8)	0.8740	0.9 (0.1-6.7)	0.8 (0.2-3.6)	0.9515	0.6 (0.2-3.1)	1 (0.2-3.6)	0.6257

Marker, MFI/Isotype (Median, range)	CD8⁺ T cell								
	ND (N: 38)	CR (N: 38)	p	CR (N: 20)	Rel (N: 20)	p	ND (N: 26)	R/R (N: 26)	p
**PD-1**	6.0 (2.8-19.4)	4.0 (2.9-14.3)	0.0012**	3.9 (2.2-10.9)	5.8 (2.4-16.5)	0.2293	7.1 (4.1-15.4)	5.8 (2.4-9.9)	0.0250*
**TIM-3**	1.8 (0.4-6.3)	1.9 (1-6.8)	0.3052	2.7 (1.4-6.8)	3.1 (1.0-5.7)	0.3591	2.2 (0.4-4.1)	2.6 (1-5.4)	0.1928
**CTLA-4**	1.1 (0.4-4.3)	1.2 (0.4-1.7)	0.4248	1.3 (0.4-2.5)	1.2 (0.4-2.3)	0.8469	1.1 (0.4-4.3)	1.2 (0.4-2.2)	0.7615
**TIGIT**	8.7 (3.7-39.8)	9.3 (3.1-35.1)	0.9153	12.3 (4.3-29.3)	10.7 (5-53.6)	0.4543	17.5 (6-56.4)	11.7 (5-38.8)	0.1591
**CD112R**	3.7 (0.9-14.3)	3.4 (1.2-8.7)	0.227	3.1 (1.2-15.7)	3.1 (1.8-5.7)	0.8469	3.4 (1.2-7.4)	3.2 (2.4-5.1)	0.1439
**CD96**	1.5 (0.4-5.1)	1.3 (0.5-5.3)	0.7114	2.6 (0.8-9.1)	3.0 (1.2-5.4)	0.7615	2.4 (0.9-5.1)	2.0 (1.0-4.3)	0.2744
**DNAM-1**	12.5 (3.2-54.4)	15.5 (6.6-52.1)	0.1502	18.2 (6.6-28.8)	18.6 (4.3-44.7)	0.5614	15.8 (3.2-73.1)	23.2 (4.3-39.4)	0.8209
**NKG2D**	2.9 (1.4-28.3)	3.7 (1.1-20.5)	0.3575	6.9 (1.9-20.5)	3.4 (2.1-23.9)	0.0034**	4.5 (0.5-11.5)	4.1 (2.1-23.9)	0.9460
**NKp30**	2.6 (0.5-5.5)	2.2 (0.7-5.9)	0.9717	1.7 (0.7-5.9)	2.2 (1.1-8.1)	0.3028	1.3 (0.5-5.5)	1.6 (0.7-8.1)	0.1928
**NKp44**	1.2 (0.4-3.8)	1.2 (0.5-3.6)	0.6782	1.9 (0.7-3.6)	1.6 (0.5-4.9)	0.4887	1.6 (0.5-3.8)	1.6 (0.5-4.9)	0.7057
**OX40**	0.8 (0.1-2.4)	0.8 (0.2-1.9)	0.9602	1.2 (0.2-4.2)	0.8 (0.1-3.8)	0.4631	0.7 (0.1-1.3)	0.8 (0.1-3.8)	0.2958

Marker, MFI/Isotype (Median, range)	Vδ1 T cell								
	ND (N: 38)	CR (N: 38)	p	CR (N: 20)	Rel (N: 20)	p	ND (N: 26)	R/R (N: 26)	p
**PD-1**	10.4 (3.3-41)	8.1 (3.7-32.6)	0.0727	10.5 (4-31.4)	9.5 (5-31.5)	0.8603	12.3 (3.3-57.2)	11.5 (5-40.2)	0.0484*
**TIM-3**	3.5 (0.7-15.7)	3.7 (1.7-10.7)	0.1954	3.7 (1.8-10.7)	3.9 (1.8-7.6)	0.2114	4.1 (0.7-13.9)	4.2 (2.5-11.1)	0.3604
**CTLA-4**	2.1 (0.6-11.4)	2.2 (0.5-7.2)	0.9154	2 (0.5-7.2)	2.4 (0-5.4)	0.4307	2.1 (0.2-6.3)	2.4 (0-5.4)	0.8736
**TIGIT**	22.6 (7.3-100.3)	34.6 (6.5-85.3)	0.7114	42.1 (6-85.3)	36.9 (17.7-130.7)	0.3225	49.1 (13.7-124.4)	36.5 (9.2-130.7)	0.0605
**CD112R**	6.9 (2.8-22)	5.8 (2.7-16)	0.2615	6.2 (2.2-35.3)	5.9 (3.6-20)	0.8501	7.3 (2.8-20.6)	6.1 (4.3-15.7)	0.4631
**CD96**	2.2 (0.4-8.2)	2.4 (0.5-49.7)	0.8736	3.9 (0.8-49.7)	3.4 (1.7-7.3)	0.2661	3.8 (0.8-7.8)	2.5 (1.5-6.5)	0.2166
**DNAM-1**	5.8 (0.9-25.2)	6.6 (1.4-145.7)	0.1689	7.9 (1.4-145.7)	9.6 (0.6-53.6)	0.8999	5.6 (1.4-65.2)	9.1 (0.6-53.6)	0.709
**NKG2D**	4.3 (1.4-26.1)	6.4 (1.2-45.1)	0.49	12.4 (3.1-31.8)	4.1 (1.3-18.7)	0.0006***	5.7 (1.9-26.1)	4.3 (1.3-18.7)	0.2455
**NKp30**	7.6 (1-35.2)	7.8 (1.2-58.5)	0.9615	5.3 (1.6-23.1)	5.8 (3.1-30)	0.7119	4.5 (1-35.2)	5.1 (2.1-22.3)	0.684
**NKp44**	2.1 (0.5-15.9)	2.2 (0.8-21.1)	0.9553	3.4 (1.7-21.1)	2.3 (1.1-11.2)	0.1754	3 (0.9-5.1)	2.3 (0.9-7.7)	0.6434
**OX40**	1.3 (0.1-28.4)	1.5 (0.1-9.3)	0.3272	1.8 (0.1-5.7)	1 (0-8.1)	0.4332	1 (0.1-3.2)	1.6 (0-8.1)	0.0583

Marker, MFI/Isotype (Median, range)	Vδ2 T cell								
	ND (N: 38)	CR (N: 38)	p	CR (N: 20)	Rel (N: 20)	p	ND (N: 26)	R/R (N: 26)	p
**PD-1**	3.3 (0.8-13.3)	2.9 (1.4-9.7)	0.3605	2.5 (1.4-6.6)	2.7 (1.2-9.1)	0.5282	3.6 (0.8-11.8)	3.5 (1.3-11.7)	0.665
**TIM-3**	4.5 (0.8-12.2)	4.5 (1.6-15.7)	0.1012	5.7 (1.6-15.7)	6.2 (1.9-30.2)	0.6685	5 (0.8-13)	5.5 (3.2-43.7)	0.2112
**CTLA-4**	2.4 (0.1-6.5)	2.2 (0.6-23.7)	0.2556	2.3 (0.8-23.7)	2.1 (0.7-4.4)	0.6685	1.9 (0.1-5.3)	2.6 (0.8-4.4)	0.2756
**TIGIT**	3.5 (0.9-12)	3.2 (0.5-25.3)	0.9158	4.3 (0.5-25.3)	3.8 (0.7-18.8)	0.6777	5 (1.6-30.4)	5.2 (0.7-15.8)	0.8334

**CD112R**	3.5 (1.1-16.2)	3.2 (1.2-7.5)	0.063	2.6 (1.2-21.6)	2.9 (0.5-5.8)	0.7869	3.2 (1.1-7.3)	3.1 (0.5-7.4)	0.8904
**CD96**	1.6 (0.5-6.7)	1.8 (0.5-35.5)	0.7454	3.6 (1.2-35.5)	2.7 (1.5-5.2)	0.4548	2.9 (1-8)	2.6 (1.2-6.7)	0.3591
**DNAM-1**	27.1 (7.7-90)	30.3 (14.4-190.1)	0.4466	42.4 (15.7-190.1)	35.9 (5.5-101.3)	0.1594	28.1 (7.7-99.4)	43.4 (5.5-101.3)	0.8996
**NKG2D**	2.5 (0.9-20.3)	4.5 (0.9-13.2)	0.3118	7.1 (1.7-16.1)	2.2 (0.8-6.9)	0.0009***	3.9 (1.3-20.3)	3.3 (0.8-6.9)	0.3554
**NKp30**	3 (0.1-11.8)	3.1 (0.4-33.5)	0.6379	3.1 (0.7-33.5)	3.4 (1-11.1)	0.7436	3.6 (0.1-11.8)	4 (1-11.7)	0.6869
**NKp44**	2.6 (0.4-7.5)	2.1 (0.3-9.5)	0.9578	2.2 (0.3-9.5)	2.3 (0.9-3.7)	0.7467	2.6 (0.4-8)	2 (0.9-3.7)	0.1074
**OX40**	1.8 (0.3-7.2)	2.8 (0.1-13.4)	0.0655	2.8 (0.1-13.4)	2.2 (0.6-6.8)	0.5016	1.4 (0.3-6.8)	1.9 (0.6-7.3)	0.3124

Supplementary Table S4. Multivariable Cox proportional hazard model for OS and RFS in T cell subsets. The increased risk for OS or RFS with increased TIM-3 expression within each T cell subset was calculated by adjusting for age, genetic risk, and the presence of secondary AML. P-values were from likelihood ratio tests. ELN: European LeukemiaNet; HR: Hazard ratio; OS: Overall survival; Ref: Reference; RFS: Relapse-free survival. $CD8^+$ T cells were defined as $CD4^-$ T cells among $CD3^+TCR\alpha\beta^+$ cells.

**Model for OS**	$CD3^+$		$CD4^+$		$CD8^+$		Vδ1		Vδ2	
**Variable**	HR	p	HR	p	HR	p	HR	p	HR	p
**TIM-3**	1.22 (1.12-1.32)	0.0002	1.09 (0.99-1.19)	0.0807	1.45 (1.22-1.76)	0.0003	1.03 (1.01-1.06)	0.0009	1.03 (1.01-1.04)	0.0047
**Age**	1.04 (1.02-1.07)	0.0015	1.02 (0.99-1.06)	0.1284	1.03 (1.00-1.06)	0.6197	1.03 (1.01-1.06)	0.0150	1.04 (1.02-1.07)	0.0023
**ELN risks**		0.0523		0.0514		0.0448		0.1060		0.0459
**Favorable**	Ref		Ref		Ref		Ref		Ref	
**Intermediate**	2.41 (0.89-6.52)		3.37 (0.73-15.50)		22.29 (1.04-244.55)		2.12 (0.78-5.77)		2.87 (1.04-7.93)	
**Adverse**	4.08 (1.44-11.57)		6.78 (1.40-32.94)		32.02 (1.12-330.18)		3.20 (1.10-9.31)		3.43 (1.22-9.62)	
**De novo AML**	0.46 (0.21-1.01)	0.0542	0.51 (0.19-1.39)	0.1755	0.57 (0.12-2.72)	0.4842	0.68 (0.32-1.45)	0.3099	0.58 (0.26-1.25)	0.1503
**Model for RFS**	$CD3^+$		$CD4^+$		$CD8^+$		Vδ1		Vδ2	
**Variable**	HR	p	HR	p	HR	p	HR	p	HR	p
**TIM-3**	1.17 (1.08-1.26)	0.0002	1.08 (0.98-1.18)	0.1317	1.31 (1.01-1.51)	0.0026	1.03 (1.01-1.05)	0.0207	1.03 (1.01-1.04)	0.0004
**Age**	1.03 (1.00-1.06)	0.0206	1.03 (0.99-1.06)	0.1111	1.03 (1.00-1.07)	0.4350	1.02 (1.00-1.04)	0.1082	1.03 (1.00-1.06)	0.0250
**ELN risks**		0.0501		0.0938		0.0381		0.1484		0.0201
**Favorable**	Ref		Ref		Ref		Ref		Ref	
**Intermediate**	2.29 (0.95-5.54)		2.60 (0.70-9.73)		8.72 (1.07-50.05)		2.09 (0.87-5.05)		3.17 (1.27-7.92)	
**Adverse**	2.81 (1.14-6.93)		4.04 (1.03-15.76)		9.10 (1.11-54.69)		2.29 (0.89-5.77)		2.70 (1.09-6.66)	
**De novo AML**	0.53 (0.26-1.09)	0.0767	0.63 (0.24-1.62)	0.3264	0.58 (0.12-2.79)	0.4962	0.72 (0.36-1.45)	0.3551	0.64 (0.32-1.28)	0.1954

Supplementary Table S5. Baseline Characteristics of All Patients and Newly Diagnosed AML Patients.

	**All patients (N: 89)**	**ND-AML only (N: 71)**
**Age at diagnosis (median, range)**	49 (19-75)	51 (19-75)
**Male (N, %)**	55 (61.8)	46 (64.8)
**WBC count, 10^9/L (median, range)**	12.2 (0.5-335.4)	17.1 (0.5-335.4)
**BM blast (%)**	76 (7-100)	76 (7-99)
***de novo* AML (N, %)**	60 (67.4)	49 (69)
**Cytogenetics (N, %)**		
RUNX1::*RUNX1T1* fusion	3 (3.4)	3 (4.2)
CBFB::*MYH11* fusion	2 (2.2)	2 (2.8)
Normal karyotype	52 (58.4)	44 (62.0)
Complex[1] karyotype	9 (10.1)	7 (9.9)
Poor karyotype[2] other than complex	3 (3.4)	1 (1.4)
Others	20 (22.5)	14 (19.7)
**Mutations (N, %)**		
FLT3-TKD	5 (5.6)	4 (5.6)
FLT3-ITD	30 (33.7)	26 (36.6)
NPM1	21 (23.6)	16 (22.5)
TP53	4 (4.5)	3 (4.2)
**ELN 2022 risks (N, %)**		
Favorable	19 (21.3)	16 (22.5)
Intermediate	32 (36)	27 (38.0)
Adverse	33 (37.1)	24 (33.8)
Undetermined	5 (5.6)	4 (5.6)
**Induction regimen (N, %)**		
IDA/ARA 3/7	66 (74.2)	52 (73.2)
DNR/ARA 3/7	23 (25.8)	19 (26.8)
**CR[3] after induction (N,%)**	52 (58.4)	40 (56.3)
Response not evaluated	1 (1,1)	1 (1.4)
**HSCT (N, %)**	64 (71.9)	51 (71.8)

1, 2, 3: According to the ELN 2022 criteria. AML, acute myeloid leukemia; ARA, cytosine arabinoside; BM, bone marrow; CR, Complete remission; DNR, daunorubicin; ELN, European LeukemiaNet; HSCT, hematopoietic stem cell transplantation; IDA, idarubicin; ND, newly diagnosed; WBC, white blood cell.
